# Construction of a High-Density Genetic Map and Analysis of Seed-Related Traits Using Specific Length Amplified Fragment Sequencing for *Cucurbita maxima*

**DOI:** 10.3389/fpls.2019.01782

**Published:** 2020-02-21

**Authors:** Yunli Wang, Chaojie Wang, Hongyu Han, Yusong Luo, Zhichao Wang, Chundong Yan, Wenlong Xu, Shuping Qu

**Affiliations:** ^1^ Key Laboratory of Biology and Genetic Improvement of Horticultural Crops (Northeast Region), Ministry of Agriculture and Rural Affairs/Northeast Agricultural University, Harbin, China; ^2^ College of Horticulture and Landscape Architecture, Northeast Agricultural University, Harbin, China

**Keywords:** *Cucurbita maxima*, high-density genetic map, specific length amplified fragment sequencing, seed-related traits, cleaved amplified polymorphic sequence

## Abstract

Seed traits are agronomically important for *Cucurbita* breeding, but the genes controlling seed size, seed weight and seed number have not been mapped in *Cucurbita maxima* (*C. maxima*). In this study, 100 F_2_ individual derived from two parental lines, “2013-12” and “9-6”, were applied to construct a 3,376.87-cM genetic map containing 20 linkage groups (LGs) with an average genetic distance of 0.47 cM using a total of 8,406 specific length amplified fragment (SLAF) markers in *C. maxima*. Ten quantitative trait loci (QTLs) of seed width (SW), seed length (SL) and hundred-seed weight (HSW) were identified using the composite interval mapping (CIM) method. The QTLs affecting SW, SL and HSW explained a maximum of 38.6%, 28.9% and 17.2% of the phenotypic variation and were detected in LG6, LG6 and LG17, respectively. To validate these results, an additional 150 F_2_ individuals were used for QTL mapping of SW and SL with cleaved amplified polymorphic sequence (CAPS) markers. We found that two major QTLs, SL6-1 and SW6-1, could be detected in both SLAF-seq and CAPS markers in an overlapped region. Based on gene annotation and non-synonymous single-nucleotide polymorphisms (SNPs) in the major SWand SL-associated regions, we found that two genes encoding a VQ motif and an E3 ubiquitin-protein ligase may be candidate genes influencing SL, while an F-box and leucinerich repeat (LRR) domain-containing protein is the potential regulator for SW in C. maxima. This study provides the first high-density linkage map of *C. maxima* using SNPs developed by SLAF-seq technology, which is a powerful tool for associated mapping of important agronomic traits, map-based gene cloning and marker-assisted selection (MAS)-based breeding in *C. maxima*.

## Introduction

The squash *Cucurbita maxima* Duch (2x = 2n = 40) is an important cucurbitaceous plant that is widely grown worldwide as a commercial crop. It has high nutritional value and health-protective properties and thus has received increasing interest and popularity in breeding. Research on squash genetics and molecular biology has also progressed rapidly in recent years.

A high-density genetic map is not only a key resource for studies on genome structure and genetic relationships but also provides the basis for quantitative trait locus (QTL) mapping and marker-assisted selection (MAS) based on the numbers of polymorphic markers (Wang et al., 2011). Since the first genetic linkage map of the genus *Cucurbita* was constructed with isozymes in 1986 (Weeden and Robinson, 1986), several genetic maps have been developed based on molecular markers. The genetic maps of *Cucurbita pepo* have been constructed with random amplified polymorphic DNA (RAPD), amplified fragment length polymorphism (AFLP), simple sequence repeat (SSR), sequence characterized amplified region (SCAR), and single-nucleotide polymorphism (SNP) markers and spanned 1954–2817.6 centimorgans (cM) (Brown and Myers, 2002; Zraidi et al., 2007; Gong et al., 2008a; Esteras et al., 2012; Monteropau et al., 2017). The high-density genetic map of *C. pepo* contained 7,718 markers with an average genetic distance between markers of 0.4 cM (Monteropau et al., 2017). The genetic maps of *Cucurbita moschata* were developed using SSR and SNP markers and spanned 1445.4–3087.03 cM (Gong et al., 2008b; Zhong et al., 2017). The high-density genetic map of *C. moschata* contained 3470 markers with an average genetic distance of 0.89 cM (Zhong et al., 2017). Compared with those of *C. pepo* and *C. moschata*, few maps of *C. maxima* with a limited number of markers have been published. The maps of *C. maxima* spanned 991.5–2566.8 cM, and the latest genetic map contained 458 markers with an average marker density of 5.60 cM (Singh et al., 2011; Ge et al., 2015; Zhang et al., 2015a). A genetic map with a low density of markers has limited application for further fine QTL mapping and MAS in squash breeding; therefore, a high-density genetic map of *C. maxima* is urgently needed.

Recently, a new high-throughput strategy for *de novo* SNP discovery called specific length amplified fragment sequencing (SLAF-seq) has been developed and successfully applied to construct high-density maps of grape (Wang et al., 2017a), gingko (Liu et al., 2017), pear (Wang et al., 2017b), tobacco (Gong et al., 2016), matsudana (Zhang et al., 2016), cauliflower (Zhao et al., 2016), soybean (Qi et al., 2014), sesame (Zhang et al., 2013), watermelon (Shang et al., 2016), and cucumber (Xu et al., 2015). This strategy has also been applied for QTL mapping of important economic and nutritional traits in sesame (Mei et al., 2017), soybean (Li et al., 2017), spinach (Qian et al., 2017), cucumber (Zhu et al., 2016), wax gourd (Jiang et al., 2015), and pumpkin (Zhong et al., 2017). However, SLAF-seq has not been successfully applied in squash. Because most genomes have characteristics of genetic variation and stability (Sun et al., 2013), SNPs are considered the most useful and conventional of all developed molecular markers. High-throughput SNP markers have potential applications for successful QTL mapping and gene cloning in *C. pepo* (Holdsworth et al., 2016; Capuozzo et al., 2017), *C. moschata* (Zhong et al., 2017) and *C. maxima* (Zhang et al., 2015b).

Seed traits are important agronomic traits for squash breeders. Larger seeds can provide more nutrients for seedlings, which can result in increased resilience against environmental stress. Many artificial-selection-based studies of squash breeding have focused on increasing seed size and weight. Most published reports related to differences in seed size have focused on the diversity and evaluation of the combining ability (Nagar et al., 2017; Darrudi et al., 2018), whereas few reports have focused on gene locations. Four seed width (SW)-associated QTLs were located using 193 F_2_ *C. maxima* individuals with logarithm of odds (LOD) values ranging from 3.49 to 4.22 and percent variance explained (PVE) values ranging from 2.87 to 29.68 cM (Tan et al., 2013). However, genes controlling seed length (SL), hundred-seed weight (HSW), and seed number per fruit (SNF) have not been mapped.

The QTLs of important seed traits in *C. maxima* have not been cloned. Therefore, a high-density linkage map with more informative and high-throughput markers is urgently needed. To construct a high-density SNP map and identify QTLs for significant seed traits in *C*. *maxima*, we used SLAF-seq technology for genotyping a population with 100 F_2_ individuals derived from two inbred lines with different agronomic traits. With this map, QTLs associated with SW, SL, SNF and HSW were successfully identified in narrow candidate regions. To locate genes controlling SL and SW, QTL mapping with cleaved amplified polymorphic sequence (CAPS) markers was performed under different growing conditions and in different populations over a two-year period. Then, the coding genes mapped in the candidate region were predicted.

## Materials and Methods

### Plant Material and DNA Extraction

An F_2_ mapping population with 100 individuals was derived from a cross between the high-generation inbred lines “2013-12” and “9-6” for SLAF-seq. An additional 150 F_2_ individuals were self-pollinated to contrast with the F_2:3_ family for locus verification using CAPS markers. Seedlings of the parental F_2_ population and F_2:3_ (10 plants per family) individuals were planted in a greenhouse at Xiangyang Base in Heilongjiang Province. Young leaves from the parents and F_2_ populations were collected for DNA extraction with the cetyltrimethylammonium bromide (CTAB) method. All fruits of the F_2_ and F_2:3_ individuals were harvested 50 days after pollination, and the seeds were cleaned and dried for seed-related phenotype analysis. The SL, SW, SNF, and HSW were measured for each offspring.

### SLAF Library Construction and High-Throughput Sequencing

An improved SLAF-seq strategy was utilized in our experiment. The *C. maxima* genome of inbred the *Cucurbita* line *C. maxima* cv. Rimu (Sun et al., 2017) was used to design SLAF markers using different enzymes. Then, a pilot SLAF experiment was performed, and the SLAF library was constructed in accordance with the predesigned scheme SLAF Predict (Peking Biomarker Technologies Corporation, Peking, China). For the F_2_ population in our study, two enzymes (HaeIII+Hpy166II) (New England Biolabs, NEB, USA) were selected as the most appropriate enzymes to digest the genomic DNA. A single-nucleotide (A) overhang was subsequently added to the digested fragments using the Klenow fragment (3´→5´ exo^-^) (NEB) and dATP at 37°C. Duplex tag-labeled sequencing adapters were ligated to the A-tailed fragments using T4 DNA ligase (ThermoFisher Scientific, MA, USA). PCR was carried out using 25-μl samples containing 100 ng of restriction-ligation DNA samples, 1× reaction buffer, 200 μM dNTPs, 0.5 U of Q5 High-Fidelity DNA Polymerase and 0.5 μM PCR primers (forward primer: 5'-AATGATACGGCGACCACCGA-3', reverse primer: 5'-CAAGCAGAAGACGGCATACG-3') (PAGE-purified, Life Technologies, China). The PCR program was as follows: 94°C for 30 s; 12 cycles of 94°C for 40 s, 65°C for 30 s, and 72°C for 30 s; and 72°C for 5 min. The products were purified using Agencourt AMPure XP beads (Beckman Coulter, High Wycombe, UK) and pooled. Next, fragments ranging from 264 bp to 364 bp in length were purified using the QIAquick Gel Extraction Kit (Qiagen, Germany). Finally, the gel-purified products were diluted to 100 ng/μl, and paired-end sequencing was performed on the Illumina HiSeq 2500 system (Illumina, Inc.; San Diego, CA, USA) according to the manufacturer's recommendations.

### Sequence Data Grouping and Genotyping

SLAF marker identification and genotyping were performed using procedures described by Sun et al. (2013). Briefly, low-quality reads (quality score < 20e) were filtered out, and then, the raw reads were sorted to each progeny according to the duplex barcode sequences. After the barcodes and terminal 5-bp positions were trimmed from each high-quality read, clean reads from the same sample filtered by SOAP software (Li et al., 2008) were mapped onto the *C. maxima* genome sequence (Sun et al., 2017). Sequences that mapped to the same position were defined as one SLAF locus (Zhang et al., 2015b). Then, SNP loci for each SLAF locus were compared between the parents; first, SLAF loci with more than 3 SNPs were filtered out. Alleles of each SLAF locus were defined according to parental reads with a sequencing depth >10-fold, whereas for each offspring, reads with sequencing depth >1-fold were used to define alleles. For pumpkin, one SLAF locus can contain at most 4 genotypes, so SLAF loci with more than four alleles were discarded subsequently. Only SLAFs with two to four alleles were identified as polymorphic markers and considered potential markers. The polymorphic SLAF loci were genotyped to ensure consistency between the parental and offspring SNP loci. Polymorphic markers were classified into eight segregation patterns. Only one segregation type (aa×bb) was used to construct the genetic map because the polymorphic markers were analyzed according to the F_2_ population type. Genotype scoring was performed using a Bayesian approach to further ensure the genotyping quality (Sun et al., 2013). Low-quality markers for each marker and each individual were counted, and the worst marker or individual was deleted. The chi-square test was performed to examine the segregation distortion, and markers with significant segregation distortion (P < 0.05) were excluded from the map construction.

### Linkage Map Construction and Evaluation

All high-quality SLAF markers were allocated into 20 linkage groups (LGs) based on their genomic locations. Then, the modified LOD (MLOD) scores between markers were calculated to further confirm the robustness of markers for each LG, and markers with MLOD scores < 5 were filtered prior to ordering. To ensure efficient construction of the high-density and high-quality map, a newly developed HighMap strategy was utilized to order the SLAF markers and correct genotyping errors within the LGs (Liu et al., 2014). First, recombinant frequencies and LOD scores were calculated using a two-point analysis and applied to infer linkage phases. Then, enhanced Gibbs sampling, spatial sampling, and simulated annealing algorithms were combined for iterative marker ordering (Jansen et al., 2001; Van, 2011). The updated recombination frequencies were used to integrate the two parental maps, which optimized the map order in the subsequent simulated annealing cycle. A stable map order was obtained after 3–4 cycles, resulting in a high-quality map including 20 LGs. The SMOOTH error correction strategy was performed according to parental contribution to the genotypes (Os et al., 2005), and a k-nearest neighbor algorithm was applied to impute missing genotypes (Huang et al., 2011). Skewed markers were added to this map by applying a multipoint maximum likelihood method. Map distances were estimated using the Kosambi mapping function (Kosambi, 1944). Marker pairs with zero recombination in each LG had the same genetic distance. The data analysis script draw haplotype-map.pl (Peking Biomarker Technologies Corporation, Peking, China) was used to construct the haplotype map, and draw heatmap.pl (Peking Biomarker Technologies Corporation, Peking, China) was used to construct the heat map.

### Relationship Between the Genetic and Physical Maps

The physical positions of the SNPs were determined based on alignment with the reference genome sequence of *C. maxima* (Sun et al., 2017). The collinearity between the genetic and physical positions was determined by plotting each marker's genetic position (in cM) against its physical position (in Mb) using Excel 2007 (Microsoft Corporation, WA, USA). Spearman's correlation coefficients were calculated using the Statistical Analysis System (SAS) program (ND Times, Peking, China).

### CAPS of DNA Polymorphisms

To verify the QTL mapping results by SLAF-seq, CAPS markers surrounding the positioning region of the major QTLs associated with SL and SW were selected (at a physical distance from 1 Mb to 5 Mb in chromosome 6). Five hundred-bp sequences surrounding SNPs from the SLAF-seq data were used to design primers for CAPS markers. The PCR products were digested by a restriction endonuclease (i.e., *EcoRI*, *HindIII*, *PstI*, *ScaI*, *BamHI*, *XhoI*) (Thermo Scientific, MA, USA) to verify polymorphisms of the CAPS markers. The primers were designed using Primer Premier 6.0 software with the appropriate CAPS candidate sequences.

The PCR mixture for CAPS amplification contained 20 ng of plant genomic DNA, 10 pmol of the primers, 0.25 mM dNTPs, 10× Taq buffer, and 1 unit of Taq polymerase in a total volume of 20 μl. Touchdown PCR was performed for 7 min at 94°C, followed by 30 cycles of 30 s at 94°C, 30 s at 60°C with stepwise decreases of 0.5°C for each cycle, and 60 s at 72°C; 10 cycles of 30 s at 94°C, 30 s at 45°C, and 60 s at 72°C; and post-heating for 7 min at 72°C. The reaction mixture for enzyme digestion contained 5 μl of the PCR product, 3.7 μl of ddH_2_O, 0.3 μl of the restriction enzyme (10 U/μl) and 1 μl of 10× enzyme buffer, which was incubated at 37°C for 3 h. The enzyme-digested products were examined *via* 2% agarose gel electrophoresis. The primer sequences for the 11 polymorphic CAPS markers are listed in **Table S1**. The enzyme-digested products were examined *via* 2% agarose gel electrophoresis at 150 V for 30 min. The agarose gel electrophoresis results were photographed using the ChampGel 6000 gel documentation and image analysis system.

### Phenotyping and QTL Mapping of Seed-Related Traits

The average SL and SW were measured for 10 randomly chosen seeds from each line with three replications, and SNF and HSW data were collected from 100 F_2_ individuals in 2017 for QTL analysis with SLAF-seq. SL and SW were measured for each individual of an additional 150 F_2_ individuals in 2017. SL and SW of 10 plants per F_2:3_ family in 2018 were measured. The means of SL and SW within each F_2:3_ family in 2018 were calculated and subjected to QTL analysis with CAPS markers.

The genotype of each mapped markers in 100 F_2_ individuals was analyzed and markers with duplicate genotypes were removed. The R package ASMap (Taylor and Butler, 2017) was used to map QTLs, and QTL analyses were then performed using the R/qtl package with the composite interval mapping (CIM) model (Churchill and Doerge, 1994). The significance of each QTL interval was tested by a likelihood-ratio statistic (LOD) (Van and Kyazma, 2004). For each trait, the LOD threshold for declaring significant QTLs was established separately with 1000 permutation tests (*P* = 0.05), which ranged from 2.8 to 3 for the various traits. To be conservative, an LOD score of 3 was used for the QTL detection of all traits. The QTLs were named according to the trait name and chromosome.

## Results

### Characteristics Between the Two Parents

**Figure 1:**
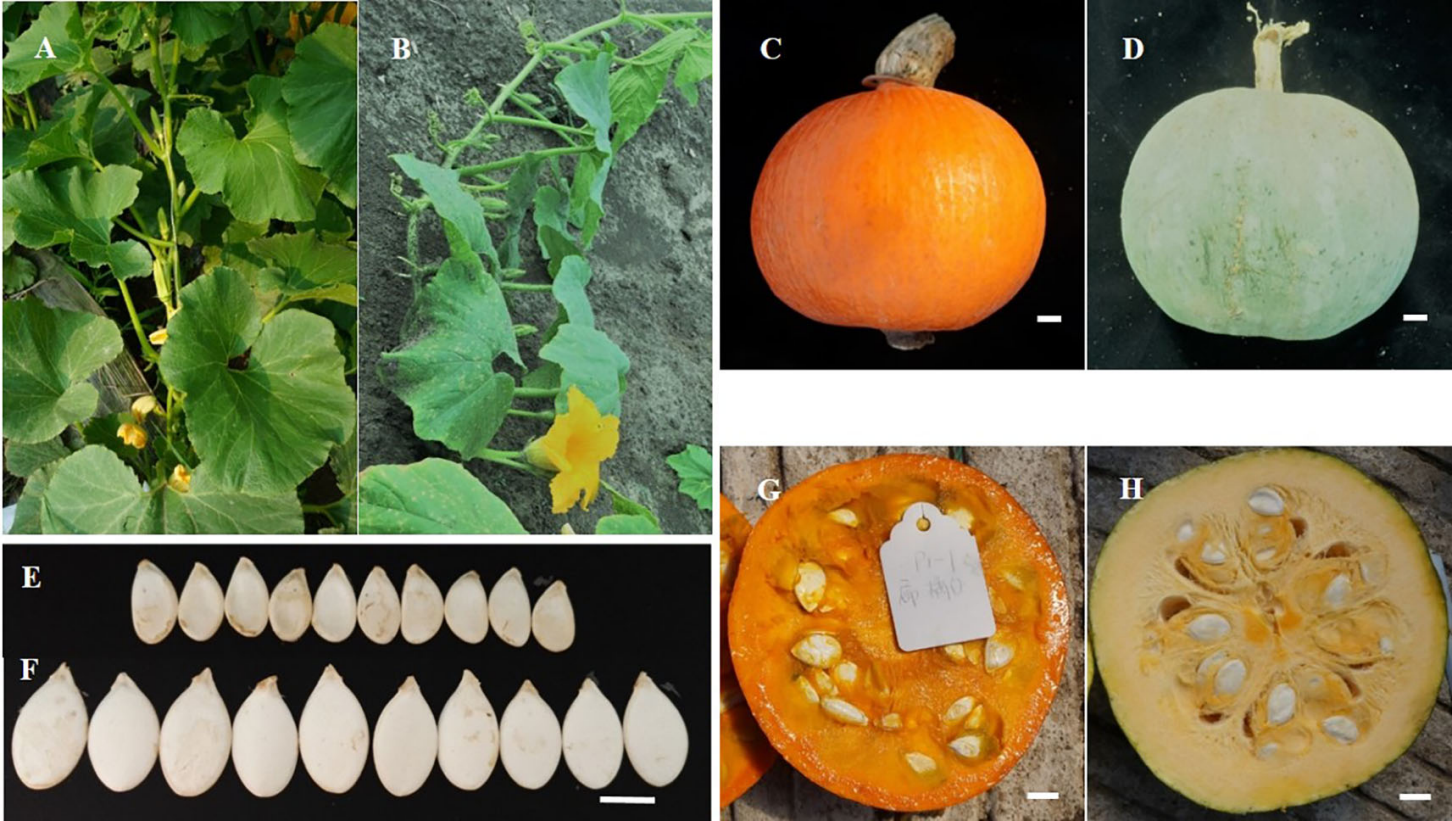
Different phenotype of two parents. A, C, E, and G are the plant type, fruit and seed phenotype of “2013-12”. B, D, F, and H are the plant, fruit and seed phenotype of “9-6”. **(A)** “2013-12” is subgynoecious type; **(B)** “9-6” is androecious type; **(C)** “2013-12” has orange-red fruits; **(D)** “9-6” has gray fruits; **(E)** “2013-12” has small and light seeds; **(F)** “9-6” has big and heavy seeds; **(G)** “2013-12” shows orang-yellow and thin flesh; **(H)** “9-6” shows light-yellow and thick flesh.

The crossing parents “2013-12” and “9-6” differed in several traits, including floral sex, fruit shape, fruit color, flesh thickness, flesh color, SL, SW, SNF, and HSW (**Figure 1** and **Table S2**). The female parent “2013-12” was subgynoecious with an orange-red fruit color, orange-yellow flesh, and small and light seeds. The male parent “9-6” was androecious with a gray fruit color, light-yellow flesh, and large and heavy seeds. The SL (20.35 mm) and SW (14.36 mm) of the F_1_ plants were similar to those of the parental “9-6” line, but the SNF (189) and HSW (36.97 g) of F_1_ were greater than those of the parental lines, with transgressions of 63.36% and 39.25%, respectively. Phenotypic data of SL, SW, SNF, and HSW were collected using 100 F_2_ individuals in 2017 for QTL mapping with SLAF-seq. Phenotypic data of SL and SW were collected using 150 F_2_ populations in 2017 and 150 F_2:3_ individuals in 2018 for QTL analysis with CAPS markers. Detailed phenotypic and genotype data of the F_2_ and F_2:3_ populations are presented in **Tables S2** and **S3**. Seed traits in 100 F_2_ individuals present continuity variance and follow the normal distribution. According to seed trait data from the parents and 100 F_2_ individuals, correlation analysis showed that SL was significantly correlated with SW (r = 0.627), which indicated that long seeds always appeared together with wide seeds.

### SLAF-Seq Data and SNP Markers

DNA sequencing generated a total of 100.4 Gb of raw bases. Each read was ∼100 bp in length and 473.35 Mb paired-end reads were obtained after SLAF library construction and high-throughput sequencing. Of these reads, 28.81 Mb reads were from the male parent, 29.41 Mb were from the female parent, and an average of 4.15 Mb were from the 100 F_2_ individuals (**Table 1**). The raw data of the two parents and 100 F_2_ individuals have been deposited to the National Center for Biotechnology Information (NCBI) and can be accessed in the Short Read Archive (SRA) sequence database under accession number PRJNA549786. Among these reads, 95.08% of the bases were of high quality, with quality scores of at least 30 (Q30). The average GC content was 40.83%, which suggested a normal distribution, and no AT or GC segregation was found in the reads. The sequencing depth of the 100 F_2_ individuals ranged from 1.61- to 6.55-fold with an average sequencing depth of 3.20-fold (**Figure S1**), which revealed that the sequencing results were reliable for marker exploration.

**Table 1:** Summary of the high-throughput sequencing data.

Sample ID	Total Reads (Mb)	Total Base (Gb)	Q30 (%)	GC (%)
2013-12	28.81	8.62	92.15	38.65
9-6	29.41	8.80	91.76	38.47
Offspring	4.15	0.83	95.14	40.88
Total	473.35	100.43	95.08	40.83

Total reads, total bases, Q30 and GC of sequencing samples are shown.

A total of 584,994 SLAFs were developed, of which 195,305 showed polymorphisms, with a polymorphism rate of 33.39% (**Table S4**). The number of SLAF markers per LG ranged from 17,334 (LG8) to 43,545 (LG4). A total of 178,376 high-quality SLAFs were detected in the offspring, of which the average sequencing depth was 29.01. In the comparison of the sequencing data from the two parents and offspring, a total of 1,437,901 SNPs were identified. Among these SNPs, the numbers of SNPs in “2013-12” and “9-6” were 690,456 and 587,028, respectively, and the average number of SNPs in each F_2_ individual was 160,417 (**Table 2**). The number of SNP markers per LG ranged from 29,881 to 77,881. SNPs with the same nucleotides for each allele depth were termed homo-SNPs. A total of 1,197,388 homo-SNPs were classified into eight segregation patterns following the CP group data format of Joinmap4.1 software (Van, 2011) and genotype encoding rules (Zhu, 2015) (**Figure S2**). The 365,639 SNPs with an aa×bb segregation pattern in the F_2_ population were selected to construct a linkage map because the F_2_ population was derived from a cross between two homozygous parents with genotype aa or bb.

**Table 2:** Statistics of SNP number.

Sample ID	SNP number	Heter-SNP number	Homo-SNP number	Heter-ratio
2013-12	690,456	233,696	456,760	33.84%
9-6	587,028	62,459	524,569	10.63%
Offspring	160,417	22,289	138,128	13.89%
Total	1,437,901	318,444	1,119,457	28.45%

SNP numbers, heter-SNP numbers, homo-SNP numbers of samples and heter-ratios of sequencing samples are shown.

### Genetic Mapping

Finally, 8,622 markers were used to construct the genetic map based on a sequencing depth in the parents of more than 10-fold, the segregation distortion criteria (P < 0.01), and less than 25% missing marker data in the F_2_ population. After removing low-collinearity markers confirmed by calculating MLOD scores between neighboring markers, 8,406 of the 8,622 SNP markers were mapped onto 20 LGs (**Table 3**, **Figure S3**). The 8,406 marker sequence and genotypes of markers in F_2_ individuals are presented in **Table S5** and **Table S6**. The genetic map spanned a total of 3376.87 cM, with an average distance of 0.47 cM between adjacent markers. The average number of markers in each LG was 420.3, and markers spanned an average length of 168.84 cM. The number of mapped SNP markers ranged from 134 (LG16) to 865 (LG11), and the average distance ranged from 0.21 cM (LG16) to 0.99 cM (LG11). LG12, which was the largest LG (196.35 cM genetic length), contained 490 markers and had an average marker density of 0.40 cM. LG9, which was the smallest LG (121.95 cM genetic length), contained 241 markers and had an average marker density of 0.51 cM. On the genetic map, the largest gap was 11.49 cM and was located in LG14. Gaps <5 cM constituted 99.30% of the total LGs, which showed good uniformity in the distribution.

**Table 3:** Basic characteristics of 20 LGs.

Linkage Group ID	Total Marker	Total Distance (cM)	Average Distance (cM)	Max Gap (cM)	Gap < 5 cM (%)
LG 01	555	187.33	0.34	10.47	99.46
LG 02	321	151.20	0.47	7.88	98.75
LG 03	430	169.59	0.40	5.42	99.53
LG 04	808	196.01	0.24	6.11	99.88
LG 05	345	171.91	0.50	6.64	99.13
LG 06	542	190.00	0.35	8.17	99.08
LG 07	280	135.77	0.49	6.55	99.64
LG 08	398	169.45	0.43	6.64	99.50
LG 09	241	121.95	0.51	7.48	99.58
LG 10	538	189.32	0.35	3.71	100.00
LG 11	865	180.77	0.21	3.71	100.00
LG 12	490	196.35	0.40	4.81	100.00
LG 13	340	170.73	0.50	5.54	99.71
LG 14	304	142.39	0.47	11.49	98.68
LG 15	305	175.14	0.58	7.86	98.68
LG 16	134	132.10	0.99	8.75	96.99
LG 17	349	193.60	0.56	8.5	99.43
LG 18	683	189.06	0.28	2.71	100.00
LG 19	245	141.37	0.58	6.03	99.18
LG 20	233	172.83	0.74	10.14	98.71
Total	8406	3376.87	0.47	11.49	99.30

Total SNP markers, total distances, average distances, maxs gap and Gap < 5 cM in each linkage group are shown. Numbers of the *C. maxima* in each LG are also shown. Gap < 5 indicated the percentages of gaps in which the distance between adjacent markers is smaller than 5 cM.

The total number of reads for the markers used to construct the genetic maps in the two parents were 134,754 and 148,231, and the average depths were 16.03 and 17.63, respectively (**Table S7**). The total marker depth in the F_2_ population ranged from 24,382 to 428,849, with a depth of 216,408 in each offspring. The average marker depth in the F_2_ population ranged from 6.11 to 51.13, with a depth of 26.24 in each offspring (**Table S8**). The high marker depth could improve the accuracy of marker locations on the genetic map.

### Evaluation of the Genetic Map

Haplotype and heat maps were used to evaluate the quality of the genetic map. Potential genotype and marker order errors can be reflected by a haplotype map of the genetic map. Haplotype maps were generated for each F_2_ individual and the parental controls using SNP markers. More double crossovers indicated more genotype and marker order errors. As shown in **Figure S4**, most regions in the haplotype map of all F_2_ individuals had a common origin, which indicated that a high-quality genetic map was constructed with the 8,406 SNPs. The relationship of recombination between markers from one LG was used to determine the potential ordering errors of the markers. Based on the heat map of the 20 LGs, a strong linkage relationship between two adjoining markers in the 20 LGs was distinctly visible (**Figure S5**). As the distance between the two markers increased, the linkage relationship between them weakened, which illustrated the correct order of markers in the LGs.

The correlation of genetic and physical positions is an important factor for evaluation of genetic maps. Spearman's correlation coefficients between the genetic and physical maps for each LG were calculated to analyze the collinear relationships between the maps (**Table S9**). Spearman's correlation coefficient of each LG ranged from 0.98 to 1, which indicated high collinearity of the genetic and physical maps. As shown in **Figure S6**, marker arrangement on the genetic map was highly consistent with that on the physical map, which indicated good collinearity of the maps and high accuracy of genetic recombination on the genetic map.

### QTL Analysis

**Figure 2:**
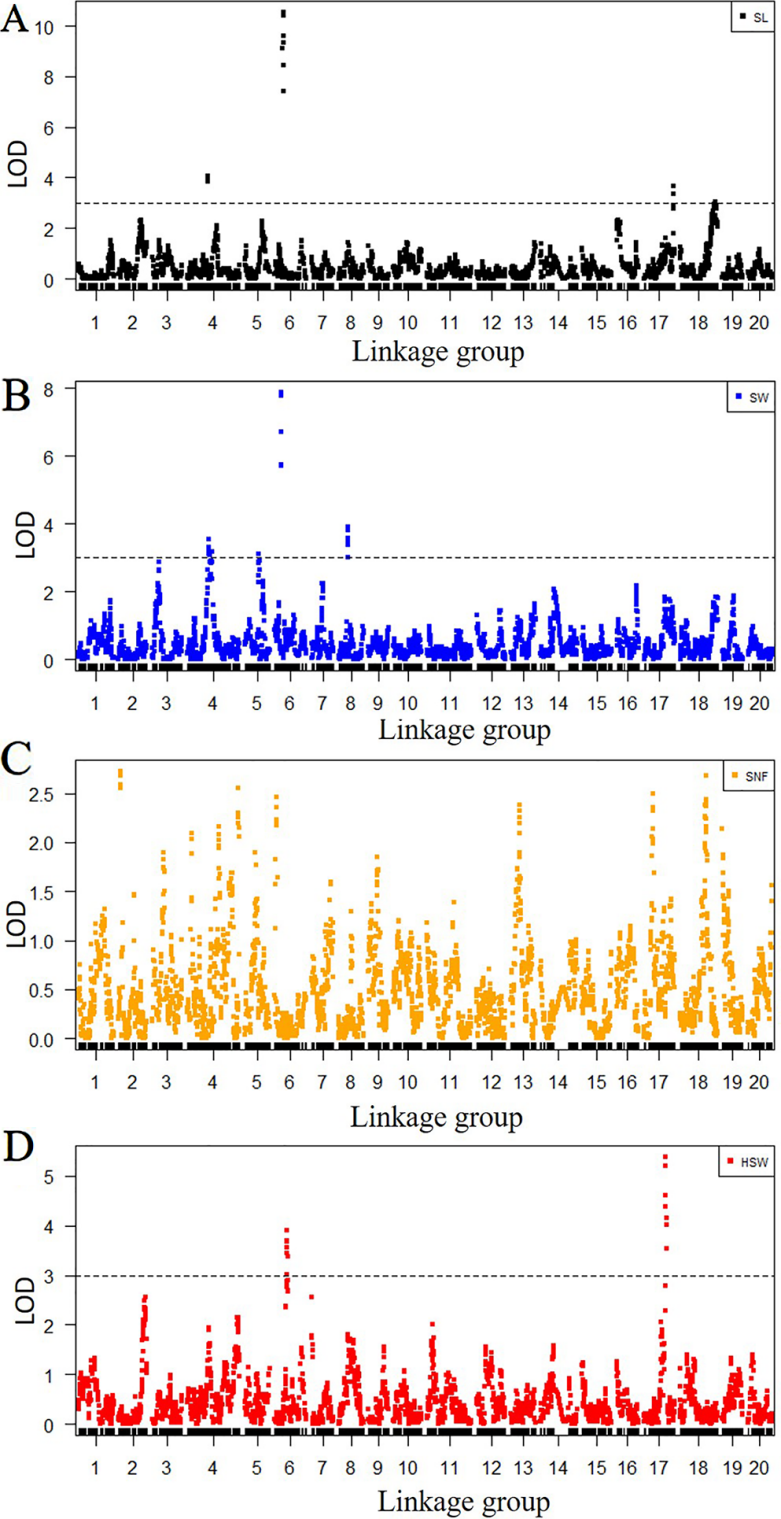
Quantitative trait locus (QTL) analysis for four seed traits according to SLAF-seq data. The x-axes indicate linkage groups, and the y-axes indicate logarithm of odds (LOD) score. **(A)** QTL analysis for SL, **(B)** QTL analysis for seed width (SW), **(C)** QTL analysis for seed number per fruit (SNF), and **(D)** QTL analysis for hundred-seed weight (HSW). The black horizontal lines on seed length (SL), SW, and HSW indicate the LOD = 3.0.

Among 8,406 markers, 2,041 markers with different genotypes were used for QTL mapping. The LGs and positions in the LGs of 2,041 markers are listed in **Table S10**. According to the genotype of the F_2_ population from the SLAF-seq data, the QTLs for SL, SW and HSW were distributed at 10 positions in six LGs, while no significant QTLs were detected in 20 LGs for SNF. The seed traits, LGs, position intervals, starting markers, ending markers, peak markers, LODs, PVEs, additive values, and dominance values are shown in **Table 4** and **Figure 2**. QTLs that could explain more than 15% of the PVEs were considered major-effect QTLs. Four QTLs were detected for SL and were located in four LGs (LG4, LG6, LG17, and LG18). These QTLs explained 7.0-38.6% of the variance. The minor-effect QTLs *SL4-1*, *SL17-1*, and *SL18-1* together explained 30.7% of the variance. The major-effect QTL *SL6-1* was situated between markers Marker157285 and Marker158784 (from 37.9 cM to 42.2 cM) with a peak at Marker158406, and explained 38.6% of the variance with an LOD value of 10.6. Four QTLs for SW were distributed over LG4, LG5, LG6, and LG8 and explained 6.9%–28.9% of the variance. The major-effect QTL *SW6-1* was situated between markers Marker154765 and Marker156546 (from 28.4 cM to 33 cM) with a peak at marker Marker155361 and explained 28.9% of the variance with an LOD value of 7.9. Three minor-effect QTLs, *SW4-1*, *SW5-1*, and *SW8-1*, together explained 30.8% of the variance. Another two QTLs for HSW were located on LG6 and LG17. One major-effect QTL, *HSW17-1*, was situated between markers Marker424927 and Marker424340 (from 99.6 cM to 111.9 cM) with a peak at marker Marker424492, and explained 17.2 of the variance with an LOD value of 5.4. In addition, *SL6-1*, *SW6-1*, and *HSW6-1* were located at the same LG.

**Table 4:** Genetic mapping and QTL analysis of 4 seed-related traits in 100 F_2_ individuals.

Trait	QTL	LG	Position interval (cM)	Start marker	End marker	Peak marker	LOD	PVE (%)	Add	Dom
Seed length	*SL4-1*	4	92.5-101.6	Marker93772	Marker94339	Marker93889	4.1	12.6	1.1	0.3
	*SL6-1*	6	37.9-42.2	Marker157285	Marker158784	Marker158406	10.6	38.6	1.6	1.3
	*SL17-1*	17	134.9-140.8	Marker421850	Marker421215	Marker421617	3.7	11.1	-0.1	-1.5
	*SL18-1*	18	143.6-176.2	Marker477731	Marker486042	Marker483115	3.0	7.0	-0.6	-0.4
Seed width	*SW4-1*	4	92.5-122.9	Marker93772	Marker96127	Marker94339	3.5	6.9	0.4	0.6
	*SW5-1*	5	60.3-88.7	Marker137649	Marker144920	Marker140172	3.1	10.9	0.6	0.5
	*SW6-1*	6	28.4-33.0	Marker154765	Marker156546	Marker155361	7.9	28.9	1.2	0.3
	*SW8-1*	8	39.6-46.9	Marker200328	Marker201237	Marker200805	3.9	13.0	0.7	0.5
Hundred seed weight	*HSW6-1*	6	53.5-64.2	Marker161001	Marker163399	Marker162384	3.9	13.2	4.2	0.6
	*HSW17-1*	17	99.6-111.9	Marker424927	Marker424340	Marker424492	5.4	17.2	0.6	-6.7

The seed traits, linkage groups, position intervals, starting markers, ending markers, peak markers, LODs, PVEs, additive values and dominance values are shown. QTLs of SL, SW, SNF and HSW are shown. LG, Linkage group; PVE, phenotype variance explained; Add, additive value; Dom, dominance value.

### Linkage Map Construction With CAPS Markers

**Figure 3:**
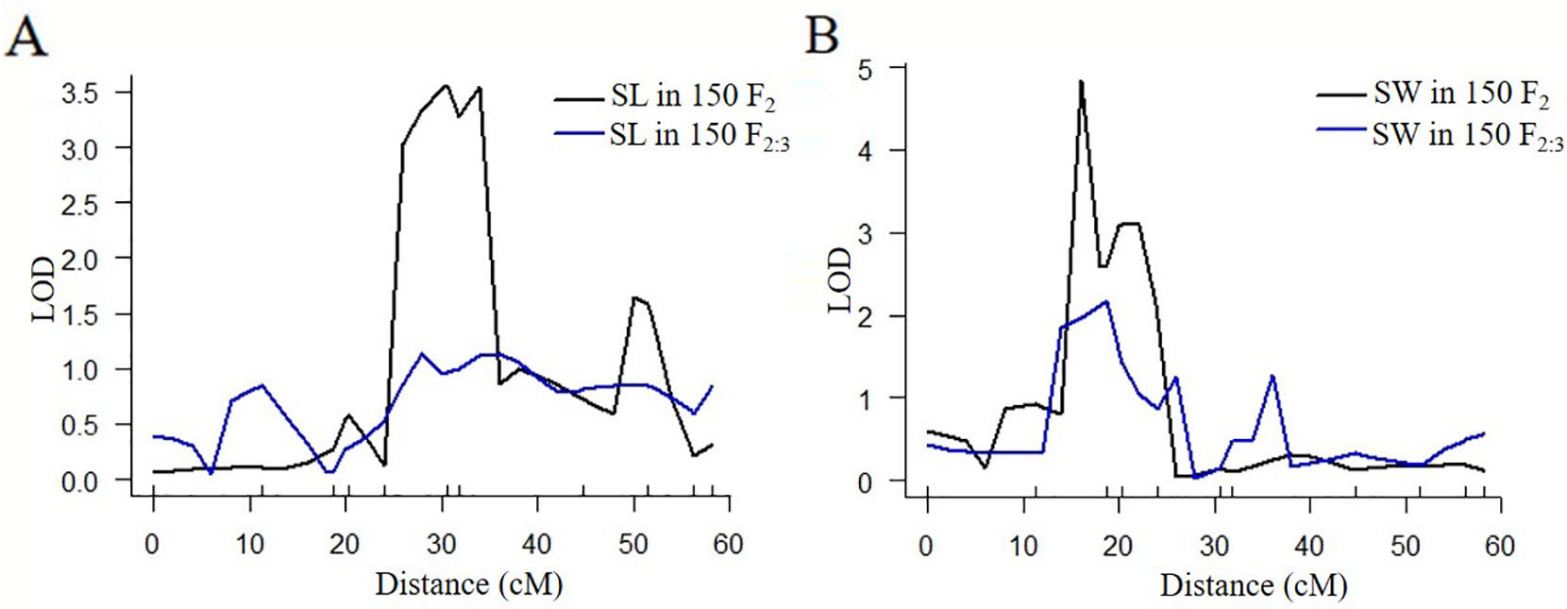
Quantitative trait locus (QTL) analysis for seed length and seed width by CAPS markers. **(A)** QTL analysis for seed length in 150 F_2_ and F_2:3_ individuals. **(B)** QTL analysis for seed width in 150 F_2_ and F_2:3_ individuals.

According to the QTL results of SLAF-seq, the major QTL of SL and SW was located at a physical location of 28.4 cM to 42.2 cM in LG6 (with a physical distance of 1.82 Mb to 2.92 Mb). To verify the location results for SL and SW, 15 pairs of CAPS markers were designed from 1.00 Mb to 5.00 Mb in LG6. Eleven pairs of the CAPS marker showed clear, repeating, polymorphic bands for “2013-12”, “9-6” and their F_1_ progeny. The 500-bp sequences surrounding SNPs for eleven CAPS markers are presented in **Table S11**. CAPS production bands consistent with “2013-12” were marked as A, those consistent with “9-6” were marked as B, and those consistent with F_1_ were marked as H. We genotyped 150 F_2_ plants with these 11 markers (**Table S3**); the LOD profiles of the QTLs for the SL and SW traits are illustrated in **Figure 3** and **Table 5**. The QTL *SL6-1F2* was situated between markers M2374213 and M3507675, with an LOD score of 3.6 and a phenotypic variation of 22.2%. The QTL *SW6-1F2* was identified between markers M1468248 and M1929605; this QTL accounted for 20.8% of the total variation and had an LOD score of 4.8. No significant QTLs were detected in the F_2:3_ population for SL and SW, while the peak regions of QTL *SL6-1F2:3* and *SW6-1F2:3* were located in regions similar to those of the peaks of *SL6-1F2* and *SW6-1F2*. The QTL *SL6-1* had an overlapping region with QTLs *SL6-1F2* at 2.52-2.92 Mb in LG6, while QTL *SW6-1* had an overlapping region with QTLs *SL6-1F2* at 1.82-1.93 Mb in LG6. These findings indicated that CAPS markers M2374213 and M3507675 could be used for MAS of the SL trait, and markers M1468248 and M1929605 could be used for MAS of SW traits.

**Table 5:** Position of major QTLs for SL and SW using CAPS markers.

QTL name	Trait	Position interval (cM)	Left marker	Right marker	Peak marker	LOD Threshold	PVE (%)	Add	Dom
*SL6-1F2*	SL	24.1-44.7	M2374213	M3507675	M2802290	3.6	22.2	-1.2	-0.1
*SL6-1F2:3*	SL	0-58.2	M1047465	M4930644	M2802290	1.1	14.6	-0.8	0.0
*SW6-1F2*	SW	11.3-18.8	M1468248	M1929605	M1929605	4.8	20.8	-0.8	0.0
*SW6-1F2:3*	SW	11.3-44.7	M1468248	M3507675	M1929605	2.2	22.2	-0.5	0.3

QTL names, position intervals, left markers and right markers, peak markers, LOD Thresholds, additive value, dominance value and PVEs are shown. ADD, additive value; DOM, dominance value; PVE, phenotype variance explained. Parent line “2013-12” account for the Add and Dom value.

### Predicted Genes for Seed Length and Seed Width

To efficiently select predicted genes controlling the SL and SW traits, the coding genes of the SL-associated region (from 2.52 Mb to 2.92 Mb) and SW-associated region (from 1.82 Mb to 1.93 Mb) in LG6 were analyzed. Protein structures and functions could be influenced by a change in a single nucleotide in the coding region. According to the SNPs and InDels from the SLAF-seq data, genes with missense codons between the two parents were selected, and twenty-two genes were identified in the candidate regions of the major QTLs for SL and SW. The gene ID and probable function information are listed in **Table 6**. Fifteen genes were annotated in SL-associated regions, and seven genes were annotated in SW-associated regions. According to seed-traits related gene descriptions (Sun et al., 2017), one gene encoding a VQ motif (*CmaCh06G005530.1*, from 2628780 bp to 2629112 bp in LG6) and one gene encoding E3 ubiquitin-protein ligase (*CmaCh06G005450*, from 2581752 bp to 2588335 bp) with a conserved domain regulates SL in other plant species (Song et al., 2007; Wang et al., 2010). Another gene encoding an F-box and leucine rich repeat (LRR) domains-containing protein (*CmaCh06G004140.1*, from 1917891 bp to 1923829 bp) with a conserved domain regulates SW in several plant species (Luo et al., 2005). The amino acid sequence alignment between “2013-12” and “9-6” for these genes is shown in **Figure S7**. The amino acid sequence differences in *CmaCh06G005450* and *CmaCh06G005530.1* between the two parental lines occurred in a conserved sequence (amino acid residues 5 to 333 aa and 14 to 75 aa, respectively). The amino acid sequence differences in *CmaCh06G004140.1* between the two parental lines occurred in anN-terminal C2 conservative domain (11-140 aa) and a structural maintenance of chromosomes (SMC) -super family conservative domain (276-1026 aa), which could affect protein function. These results indicate that *CmaCh06G005450*, *CmaCh06G005530.1*, and *CmaCh06G004140.1* might be good candidates to explain differences in SL and SW between the parents and offspring. Further experiments are needed to confirm the functions of these genes in *Cucurbita*.

**Table 6:** The predicted genes in major QTLs for SL and SW.

QTL name	Trait	Gene ID	Gene description
*SL6-1*	Seed Length	*CmaCh06G005340.1*	CSL zinc finger domain-containing protein
		*CmaCh06G005350.1*	MD-2-related lipid recognition domain-containing protein
		*CmaCh06G005360.1*	protein disulfide oxidoreductase
		*CmaCh06G005400.1*	Polyketide cyclase/dehydrase and lipid transport superfamily protein
		*CmaCh06G005450.1*	E3 ubiquitin-protein ligase RHF2A
		*CmaCh06G005480.1*	Sucrose-6F-phosphate phosphohydrolase
		*CmaCh06G005500.1*	ABC transporter G family member 22-like
		*CmaCh06G005530.1*	VQ motif
		*CmaCh06G005540.1*	Zein-binding
		*CmaCh06G005620.1*	protein downstream neighbor of Son isoform X1
		*CmaCh06G005640.1*	homogentisate phytyltransferase 2
		*CmaCh06G005780.1*	Ferredoxin–NADP reductase
		*CmaCh06G005820.1*	Beta-carotene isomerase D27
		*CmaCh06G005890.1*	Serine/threonine protein kinase
		*CmaCh06G005920.1*	N6-adenosine-methyltransferase MT-A70-like
*SW6-1*	Seed width	*CmaCh06G003930.1*	Lipid A export ATP-binding/permease protein MsbA
		*CmaCh06G003980.1*	Xyloglucan endotransglucosylase/hydrolase
		*CmaCh06G004000.1*	ACT domain-containing family protein
		*CmaCh06G004090.1*	UDP-Glycosyltransferase superfamily protein
		*CmaCh06G004100.1*	UDP-Glycosyltransferase superfamily protein
		*CmaCh06G004120.1*	Acyl-CoA N-acyltransferase with RING/FYVE/PHD-type zinc finger domain-containing protein
		*CmaCh06G004140.1*	F-box and Leucine Rich Repeat domains containing protein

Seed traits, gene IDs and gene descriptions of QTL are shown.

## Discussion

SL, SW, SNF, and HSW have significant effects on squash yield and seed quality. Breeders prefer high seed yields and heavy seeds. The F_1_ plants derived from a cross between the “2013-12” line and “9-6” lines exhibited over-parent heterosis in SNF and HSW. The “9-6” line could provide a new allele source for pumpkin breeding with large and heavy seeds.

High-density genetic maps are a highly valuable tool for map-based cloning and MAS. This study reports the construction of the first high-density linkage map of *C. maxima* using SNPs identified by SLAF-seq technology. When we compared the sequencing data from the two parents and offspring, a total of 584,994 SLAF markers and 1,437,901 SNPs were identified, and the average sequencing depth was 29.01. A number of markers will be beneficial for gene determinations and molecular cloning in pumpkin breeding. The genetic map studied herein spanned a total of 3,376.87 cM, and the linkage map in our study exhibited 20 LGs with approximately the same physical distances as the reference genome (Sun et al., 2017). It was indicated that the genetic map and SLAF markers in our study were of good quality. A total of 8,406 SNPs were used to construct a genetic map in 20 LGs with an average distance of 0.47 cM between adjacent markers, which meant that more markers were identified here than for *C. maxima* with 458 bin markers (Zhang et al., 2015b), *C. moschata* with 3,470 SNPs (Zhong et al., 2017) and *C. pepo* with 7,718 SNPs (Monteropau et al., 2017). The mean distance between markers in the genetic map in our study was shorter than those for *C. maxima,* with a mean marker density of 5.60 cM (Zhang et al., 2015b), and *C. moschata,* with a mean marker density of 0.89 cM (Zhong et al., 2017), and was almost as short as that for *C. pepo,* with a mean marker density of 0.4 cM (Monteropau et al., 2017). The published high-density genetic maps of *Cucurbita* were constructed using genotyping-by-sequencing (GBS) (Zhang et al., 2015b; Monteropau et al., 2017) and double-digest restriction-associated DNA (ddRAD) libraries (Zhong et al., 2017). GBS and ddRAD genotyping is performed by randomly interrupting genomic DNA with restriction enzymes, whereas SLAF-seq is performed by sequencing the paired ends of sequence-specific restriction fragments (Xu et al., 2015; Zhang et al., 2015b; Zhong et al., 2017). Therefore, SLAF-seq provides better repeatability and deeper sequencing than the above methods.

High-density markers and sites are necessary for map-based cloning of genes. In the genetic map of our study, LG9 was the largest LG (196.35 cM) and contained 490 markers, whereas LG12 was the smallest (121.95 cM) and contained 241 markers. On the map of *C. maxima* published by Zhang et al. (2015b), the smallest LG was LG10 (47.61 cM), which harbored 10 markers on 1 oriented scaffold, and the largest LG was LG4 (268.00 cM), which harbored 41 markers on 2 oriented scaffolds. These two genetic maps of *C. maxima* showed little consistency in LG length. The evenly distributed SNPs and high-density sites studied herein were developed with SLAF-seq and HighMap methods. The haplotype map, heat map and Spearman correlation coefficients of the genetic map further ensured the SNP location accuracy in the LGs.

On the published high-density linkage map of *C. maxima*, the distance between neighboring markers ranged from 0.05 cM to 44.89 cM, and 8 large gaps (≥18 cM) were detected in LG2, LG3, LG6, LG7, LG11, LG15, and LG19 (Zhang et al., 2015b). On the published high-density linkage map of *C. moschata*, the largest interval gap was 22.30 cM, and large gaps were detected in LG8, LG13, LG16, and LG19 (Zhong et al., 2017). The large gaps and low sequencing coverage have limited further fine mapping and MAS in *Cucurbita*. Therefore, additional markers and high-coverage genetic maps are needed. In our study, the largest interval gap was only 11.49 cM (in LG14), and gaps <5 cM constituted up to 99.30% of the total LG, which illustrated successful construction of a map with improved coverage. There may be two possible explanations for the high-coverage genetic map. First, the two elite parents used for this map presented significant differences in several traits (**Figure 1**), which indicated the existence of great genetic diversity between the parents. Second, to obtain SLAF markers with few repeated sequences that are evenly distributed in 20 LGs, the squash genome was used to design marker discovery experiments using different enzymes. Two enzymes were used to digest the genomic DNA in this experiment, which ensured that SLAF markers were effectively discovered on a genomic map.

Based on differences in seed trait composition between the two parents in our study, we identified a total of ten QTLs for SL, SW and HSW with the CIM method. Four QTLs were found for SL, four QTLs were found for SW, and two QTLs were found for HSW in our study. No significant QTLs for SNF were detected in our study, most likely because of a slight difference between the parents “2013-12” (116 SNF) and “9-6” (105 SNF). In cucumber and watermelon, 12 QTLs were found for SL with PVE values ranging from 2.20% to 28.90%, 12 QTLs for SW with PVE values ranging from 2.24% to 24.10%, and 10 QTLs for HSW with PVE values ranging from 4.50% to 28.30% (Wang et al., 2014; Zhou et al., 2016). In our study, major QTL *SL6-1* was homologous to Chr3, with a physical distance of 28.29 Mb-32.43 Mb in cucumber (Qi et al., 2013), which is near the SSR22874 marker of *SL3.1* (at a physical distance of 34.08 Mb) in cucumber (Wang et al., 2014). These results indicated that SL is controlled by homologous genes among species. Homologous regions of major QTLs for SW and HSW in cucumber or watermelon (Zhou et al., 2016) were located on different chromosomes or in different locations, in contrast to published QTLs in cucumber and watermelon (Wang et al., 2014; Zhou et al., 2016). Thus, multiple genes may control SW and HSW in different cucurbit species.

According to the seed size data, we found that long seeds always appeared together with wide seeds in the offspring in our study (**Table S2**). Further analysis showed that SL was significantly correlated with SW. The QTL of *SL-1* was in a region similar to that of *SW6-1* in the same LG, resulting in similar colocalization between SL and SW. It would benefit breeding for large seeds. In published studies of seed traits, the major QTLs for SL and SW in watermelon were colocalized in the same LG (Meru and McGregor, 2013; Zhou et al., 2016), which was similar to our results. Four significant QTLs for SL and four significant QTLs for SW were identified, and the flanking markers for these QTLs could be used in molecular MAS to increase the seed size in squash seedlings during the selection of hybrid offspring.

Quantitatively inherited traits can be influenced by the environment and mapping population. In our study, we detected seed size traits (seed length and seed width) with 100 F_2_ populations by the CIM method from SLAF-seq data in 2017 (the related QTLs were named *SL4-1* to SL17-1 and *SW5-1* to *SW8-1*), 150 F_2_ populations by CAPS markers in 2017 (the QTLs were named *SL6-1F2* and *SW6-1F2*), and 150 F_2:3_ individuals by CAPS markers in 2018 (the QTLs were named *SL6-1F2:3* and *SW6-1F2:3*).The peaks of QTLs *SL6-1F2* and *SL6-1F2:3* were collected from the same marker, and the peaks of QTLs *SW6-1F2* and *SW6-1F2:3* were also collected from the same marker. The major QTL *SL6-1* mapped with 100 F_2_ individuals had an overlapping region with *SL6-1F2* mapped with additional 150 F_2_ individuals, indicating that this region had high LOD scores and PVE values in different individuals from the same year. On the other hand, the QTL *SL6-1F2:3* mapped with 150 F_2:3_ individuals had a low LOD value, which might have been influenced by the different growing environments in the different years. Similar conditions were found in SW QTLs. The overlapped region of *SL6-1* and *SL6-1F2* (from 2.52 Mb to 2.92 Mb) was the SL-associated region, and the overlapped region of *SW6-1* and *SW6-1F2* (from 1.82 Mb to1.93 Mb) was the SW-associated region. Closely linked CAPS markers (M2374213, M3507675, M1468248, and M1929605) could be used for MAS-based breeding in *C. maxima* for seed elongation and the radius. With regard to the available SLAF-seq data, crossed parents with differences in multiple phenotypes were used in our study, and the QTLs controlling important agronomic traits were mapped. Only 100 F_2_ individuals were used to construct the SLAF library and map complex seed-related traits in *C. maxima*, and accurate QTL mapping results were obtained. Therefore, SLAF-seq is a useful gene mapping technology for small populations and exhibits a high success rate and stability.

The early growth of endosperm is coordinated with the seed coat growth and plays a direct role in determining the ripened seed size (Garcia et al., 2005; Ingram, 2010). The seed size of *Arabidopsis* is regulated by the *IKU-MINI* pathway, which is thought to control endosperm growth. The *IKU-MINI* pathway includes *IKU1*, which encodes a protein containing a VQ motif; and *MINI3,* which encodes a WRKY transcription factor (Wang et al., 2010). *IKU2* also encodes an LRR kinase and shows a recessive mode of action causing reduction in endosperm growth accompanied by precocious cellularization and reduced seed size in *Arabidopsis* (Luo et al., 2005). In the SL-associated region, we found *CmaCh06G005530.1*, which encodes a protein containing a VQ motif. We found one amino acid difference in *CmaCh06G005530.1* between the two parental lines in a conserved sequence (**Figure S7B**). In the SW-associated region, *CmaCh06G004140*, encoding an F-box and LRR domain-containing protein, had several amino acid differences between the two parental lines in a conserved domain of SMC, which controls the cell cycle control and cell division (**Figure S7C**). These findings suggested that *CmaCh06G005530.1* and *CmaCh06G004140* were the candidate genes responsible for seed length and width, and the seed size of *C. maxima* was also regulated by the *IKU1* and *IKU2* pathway. One gene, *GW2*, encoding a protein with E3 ubiquitin ligase activity, was found to affect the grain size, weight and yield by controlling the endosperm size in rice (Song et al., 2007). Loss of *GW2* function could increase cell numbers, resulting in increased spikelet hull size and enhanced grain size. In the SL-associated region, *CmaCh06G005450* was selected, which encodes E3 ubiquitin-protein ligase. One amino acid difference in *CmaCh06G005450* between the two parental lines was observed in a conserved sequence (**Figure S7A**). This finding indicated that the *CmaCh06G005450* gene might affect seed size, which is thought to control cell numbers in our *Cucurbita* materials. The SNP information from SLAF-seq was limited compared to the whole-genome resequencing results, and some regions within the QTL interval were not sequenced. Coding genes without nonsynonymous SNPs in major QTL regions were also considered candidate genes. CAPS markers M2374213, M3507675, M1468248, and M1929605 were used for single segment substitution line (SSSL) construction. Based on it, further fine mapping of SL and SW genes and gene expression during the seed development stage will be performed in our laboratory.

## Conclusion

By using 100 F_2_ populations from two morphologically diverse parents, a high-density genetic map of *C. maxima* was constructed by SLAF-seq. With this map, 10 QTLs for seed-related traits were identified, and major QTLs for SW, SL, and HSW were detected in *C. maxima* for the first time. An additional 150 F_2_ and F_2:3_ populations were used to map SW and SL with CAPS markers, and the results indicated that the QTL mapping using SLAF-seq was accurate. Nonsynonymous SNPs and gene descriptions of the SL- and SW-associated regions suggested that the genes encoding a VQ motif gene, E3 ubiquitin-protein ligase and an F-box and LRR domain-containing protein might be associated with SW and SL in *C. maxima*. In summary, the high-density genetic map studied herein is a valuable tool for association mapping of important agronomic traits, map-based gene cloning and MAS breeding in *Cucurbita*.

## Data Availability Statement

The datasets generated for this study can be found in the the National Center for Biotechnology Information (NCBI), accession number PRJNA549786.

## Author Contributions

YW performed data analysis, preparing the manuscript. CW contributed to collecting phenotypic characteristics and DNA extraction. HH contributed to CAPS demonstration test. WX and ZW contributed to growing plants. YL and CY contributed to providing experimental material. SQ, the corresponding author, oversaw all activities related to the project implementation and manuscript development.All authors read and approved the final version of the manuscript.

## Funding

This work was supported by grants from the National Key Research and Development Program of China (2018YFD0100706), the Natural Science Foundation of Heilongjiang Province of China (C2018027), and the Youth Foundation of Northeast Agricultural University (17QC08).

## Conflict of Interest

The authors declare that the research was conducted in the absence of any commercial or financial relationships that could be construed as a potential conflict of interest.

## References

[B1] Brown R. N. Myers J. (2002). A genetic map of squash (*Cucurbita* sp.) with randomly amplified polymorphic DNA markers and morphological markers. J. Am. Soc. Hort. Sci. 127 (4), 568–575. 10.21273/JASHS.127.4.568

[B2] Capuozzo C. Formisano G. Iovieno P. Andolfo G. Tomassoli L. Barbella M. M. (2017). Inheritance analysis and identification of SNP markers associated with ZYMV resistance in *Cucurbita pepo* . Mol. Breed. 37 (8), 99. 10.1007/s11032-017-0698-5

[B3] Churchill G. A. Doerge R. W. (1994). Empirical threshold values for quantitative trait mapping. Genetics 138, 963–971. 10.1101/gad.8.21.2653 7851788 PMC1206241

[B4] Darrudi R. Nazeri V. Soltani F. Shokrpour M. Ercolano M. R. (2018). Evaluation of combining ability in *Cucurbita pepo* L. and *Cucurbita moschata* Duchesne accessions for fruit and seed quantitative traits. J. Appl. Res. Med. Aromatic Plants 9, 70–77. 10.1016/j.jarmap.2018.02.006

[B5] Esteras C. Gomez P. Monforte A. Blanca J. Dolera N. V. Roig C. (2012). High-throughput SNP genotyping in *Cucurbita pepo* for map construction and quantitative trait loci mapping. BMC Genom. 13 (1), 80–102. 10.1186/1471-2164-13-80 PMC3359225 22356647

[B6] Garcia D. Fitz Gerald J. N. Berger F. (2005). Maternal control of integument cell elongation and zygotic control of endosperm growth are coordinated to determine seed size in *Arabidopsis* . Plant Cell 17 (1), 52–60. 10.1105/tpc.104.027136 15598800 PMC544489

[B7] Ge Y. Li X. Yang X. X. Cui C. S. Qu S. P. (2015). Genetic linkage map of *Cucurbita maxima* with molecular and morphological markers. Genet. Mol. Res. 14 (2), 5480–5484. 10.4238/2015.May.22.18 26125744

[B8] Gong L. Stift G. Kofler R. Pachner M. Lelley T. (2008a). Microsatellites for the genus Cucurbita and SSR-based genetic linkage map of *Cucurbita pepo* L. Theoret. Appl. Genet. 117 (1), 37–48. 10.1007/s00122-008-0750-2 18379753 PMC2413107

[B9] Gong L. Pachner M. Kalai K. Lelly T. (2008b). SSR-based genetic linkage map of *Cucurbita moschata* and its synteny with *Cucurbita pepo* . Genome 51 (11), 878–889. 10.1139/G08-072 18956020

[B10] Gong D. Huang L. Xu X. Wang C. Ren M. (2016). Construction of a high-density SNP genetic map in flue-cured tobacco based on SLAF-seq. Mol. Breed. 36 (7), 100. 10.1007/s11032-016-0514-7

[B11] Holdsworth W. L. LaPlant K. E. Bell D. C. Jahn M. M. Mazourek M. (2016). Cultivar-based introgression mapping reveals wild species-derived *Pm-0*, the major powdery mildew resistance locus in squash. Plos One 11 (12), e0167715. 10.1371/journal.pone.0167715 27936008 PMC5147965

[B12] Huang X. Zhao Y. Wei X. Li C. Wang A. Zhao Q. (2011). Genome-wide association study of flowering time and grain yield traits in a worldwide collection of rice germplasm. Nature Genet. 44 (1), 32–39. 10.1038/ng.1018 22138690

[B13] Ingram G. C. (2010). Family life at close quarters: communication and constraint in angiosperm seed development. Protoplasma 247 (3-4), 195–214. 10.1007/s00709-010-0184-y 20661606

[B14] Jansen J. ADe J. Ooijen J. W. V. (2001). Constructing dense genetic linkage maps. Theoret. Appl. Genet. 102 (6-7), 1113–1122. 10.1007/s001220000489

[B15] Jiang B. Liu W. Xie D. Peng Q. He X. Lin Y. (2015). High-density genetic map construction and gene mapping of pericarp color in wax gourd using specific-locus amplified fragment (SLAF) sequencing. BMC Genom. 16 (1), 1035. 10.1186/s12864-015-2220-y PMC4673774 26647294

[B16] Kosambi D. (1944). The estimation of map distances from recombination values. Annals Human Genet. 12 (1), 172–175. 10.1111/j.1469-1809.1943.tb02321.x

[B17] Li R. Li Y. Kristiansen K. Wang J. (2008). SOAP: short oligonucleotide alignment program. Bioinformatics 24 (5), 713–714. 10.1093/bioinformatics/btn025 18227114

[B18] Li B. Fan S. Yu F. Chen Y. Zhang S. Han F. (2017). High−resolution mapping of QTL for fatty acid composition in soybean using specific locus amplified fragment sequencing. Theoret. Appl. Genet. 130 (7), 1467–1479. 10.1007/s00122-017-2902-8 28389769 PMC5487593

[B19] Liu D. Y. Ma C. X. Hong W. G. Huang L. Liu M. Liu H. (2014). Construction and analysis of high-density linkage map using high-throughput sequencing data. Plos One 9 (6), e98855. 10.1371/journal.pone.0098855 24905985 PMC4048240

[B20] Liu H. Cao F. Yin T. Chen Y. (2017). A highly dense genetic map for Ginkgo biloba constructed using sequence-based markers. Front. Plant Sci. 8, 1041–1050. 10.3389/fpls.2017.01041 28663754 PMC5471298

[B21] Luo M. Dennis E. S. Berger F. Peacock W. J. Chaudhury A. (2005). *MINISEED3* (*MINI3*), a WRKY family gene, and *HAIKU2* (*IKU2*), a leucine-rich repeat (LRR) kinase gene, are regulators of seed size in *Arabidopsis* . Proceed. Nat. Acad. Sci. U. S. A. 102 (48), 17531–17536. 10.1073/pnas.0508418102 PMC1297679 16293693

[B22] Mei H. Liu Y. Du Z. Wu K. Cui C. Jiang X. (2017). High-density genetic map construction and gene mapping of basal branching habit and flowers per leaf axil in sesame. Front. Plant Sci. 8, 636–646. 10.3389/fpls.2017.00636 28496450 PMC5406510

[B23] Meru G. McGregor C. (2013). Genetic mapping of seed traits correlated with seed oil percentage in watermelon. Hort. Sci. 48 (8), 955–959. 10.1007/s13580-013-0059-1

[B24] Monteropau J. Blanca J. Esteras C. Martínezpérez E. M. Gómez P. Monforte A. J. (2017). An SNP-based saturated genetic map and QTL analysis of fruit-related traits in Zucchini using Genotyping-by-sequencing. BMC Genom. 18 (1), 94. 10.1186/s12864-016-3439-y PMC5241963 28100189

[B25] Nagar A. Sureja A. K. Munshi A. D. Bhardwaj R. Kumar S. Tomar B. S. (2017). Heritability, correlation and genetic divergence for different seed traits in pumpkin (*Cucurbita moschata*). Indian J. Agri. Sci. 87 (11), 1519–1523.

[B26] Os H. V. Stam P. Visser R. G. F. Eck H. J. V. (2005). SMOOTH: a statistical method for successful removal of genotyping errors from high-density genetic linkage data. Theoret. Appl. Genet. 112 (1), 187–194. 10.1007/s00122-005-0124-y 16258753

[B27] Qi J. Liu X. Shen D. Miao H. Xie B. Li X. (2013). A genomic variation map provides insights into the genetic basis of cucumber domestication and diversity. Nature Genet. 45, 1510–1515. 10.1038/ng.2801 24141363

[B28] Qi Z. Huang L. Zhu R. Xin D. Liu C. Han X. (2014). A high-density genetic map for soybean based on specific length amplified fragment sequencing. Plos One 9 (8), e104871. 10.1371/journal.pone.0104871 25118194 PMC4130620

[B29] Qian W. Fan G. Y. Liu D. D. Zhang H. L. Wang X. W. Wu J. (2017). Construction of a high-density genetic map and the X/Y sex-determining gene mapping in spinach based on large-scale markers developed by specific-locus amplified fragment sequencing (SLAF-seq). BMC Genom. 18 (1), 276–286. 10.1186/s12864-017-3659-9 PMC5379770 28376721

[B30] Shang J. Li N. Li N. Xu Y. Ma S. Wang J. M. (2016). Construction of a high-density genetic map for watermelon (*Citrulluslanatus* L.) based on large-scale SNP discovery by specific length amplified fragment sequencing (SLAF-seq). Sci. Hort. 203, 38–46. 10.1016/j.scienta.2016.03.007

[B31] Singh A. K. Singh R. Weeden N. F. Robinson R. W. Singh N. K. (2011). A linkage map for *Cucurbita maxima* based on randomly amplified polymorphic DNA (RAPD) markers. Indian J. Hort. 68 (1), 44–50. 10.1016/j.scienta.2011.01.011

[B32] Song X. J. Huang W. Shi M. Zhu M. Z. Lin H. X. (2007). A QTL for rice grain width and weight encodes a previously unknown RING-type E3 ubiquitin ligase. Nature Genet. 39 (5), 623–630. 10.1038/ng2014 17417637

[B33] Sun X. W. Liu D. Y. Zhang X. F. Li W. B. Liu H. Hong W. G. (2013). SLAF-seq: an efficient method of large-scale *de novo* SNP discovery and genotyping using high-throughput sequencing. Plos One 8 (3), e58700. 10.1371/journal.pone.0058700 23527008 PMC3602454

[B34] Sun H. Wu S. Zhang G. Jiao C. Guo S. Ren Y. (2017). Karyotype Stability and Unbiased Fractionation in the Paleo-Allotetraploid Cucurbita Genomes. Mol. Plant 10 (10), 1293–1306. 10.1016/j.molp.2017.09.003 28917590

[B35] Tan X. Z. Ge Y. Xu W. L. Cui C. S. Qu S. P. (2013). Construction of genetic linkage map and QTL analysis for seed width in pumpkin (*Cucurbita maxima*). Acta Bot. Boreali-Occidentalia Sinica 33 (4), 697–702.

[B36] Taylor J. Butler D. (2017). R package ASMap: efficient genetic linkage map construction and diagnosis. J. Stat. Softw. 79 (6), 1–29. 10.18637/jss.v079.i06 30220889

[B37] Van O. J. Kyazma B. V. (2004). MapQTL 5, Software for the mapping of quantitative trait loci in experimental populations. Plant Res. Int. Wageningen, Netherlands.

[B38] Van O. J. (2011). Multipoint maximum likelihood mapping in a full-sib family of an outbreeding species. Genet. Res. 93 (5), 343–349. 10.1017/S0016672311000279 21878144

[B39] Wang A. Garcia D. Zhang H. Feng K. Chaudhury A. Berger F. (2010). The VQ motif protein IKU1 regulates endosperm growth and seed size in *Arabidopsis* . Plant J. 63 (4), 670–679. 10.1111/j.1365-313X.2010.04271.x 20545893

[B40] Wang J. Lydiate D. J. Parkin I. A. Falentin C. Delourme R. Carion P. W. (2011). Integration of linkage maps for the *Amphidiploid Brassica napus* and comparative mapping with *Arabidopsis* and *Brassica rapa* . BMC Genom. 12 (1), 101–121. 10.1186/1471-2164-12-101 PMC3042011 21306613

[B41] Wang M. Miao H. Zhang S. P. Liu S. L. Dong S. Y. Wang Y. (2014). Inheritance analysis and QTL mapping of cucumber seed size. Acta Hort. Sinica 41 (1), 63–72. 10.16420/j.issn.0513-353x.2014.01.010

[B42] Wang J. Su K. Guo Y. Xing H. Zhao Y. Liu Z. (2017a). Construction of a high-density genetic map for grape using specific length amplified fragment (SLAF) sequencing. Plos One 12 (7), e0181728. 10.1371/journal.pone.0181728 28746364 PMC5528875

[B43] Wang L. Li X. Wang L. Xue H. Wu J. Yin H. (2017b). Construction of a high-density genetic linkage map in pear (*Pyrus communis* × *Pyrus pyrifolia nakai*) using SSRs and SNPs developed by SLAF-seq. Sci. Hort. 218, 198–204. 10.1016/j.scienta.2017.02.015

[B44] Weeden N. F. Robinson R. W. (1986). Allozyme segregation ratios in the interspecific cross *Cucurbita maxima* × C. *ecuadorensis* suggest that hybrid breakdown is not caused by minor alteration in chromosome structure. Genetics 114 (2), 593–609. 17246350 10.1093/genetics/114.2.593 PMC1202959

[B45] Xu X. Xu R. Zhu B. Yu T. Qu W. Lu L. (2015). A high-density genetic map of cucumber derived from specific length amplified fragment sequencing (SLAF-seq). Front. Plant Sci. 5 (768), 768–776. 10.3389/fpls.2014.00768 25610449 PMC4285734

[B46] Zhang Y. Wang L. Xin H. Li D. Ma C. Ding X. (2013). Construction of a high-density genetic map for sesame based on large scale marker development by specific length amplified fragment (SLAF) sequencing. BMC Plant Biol. 13 (1), 141. 10.1186/1471-2229-13-141 24060091 PMC3852768

[B47] Zhang J. Zhang Q. X. Cheng T. R. Yang W. R. Pan H. T. Zhong J. J. (2015a). High-density genetic map construction and identification of a locus controlling weeping trait in an ornamental woody plant (*Prunus mume Sieb.* et Zucc). DNA Res. 22 (3), 183–192. 10.1093/dnares/dsv003 25776277 PMC4463843

[B48] Zhang G. Ren Y. Sun H. Guo S. Zhang F. Zhang J. (2015b). A high-density genetic map for anchoring genome sequences and identifying QTLs associated with dwarf vine in pumpkin (*Cucurbita maxima* Duch.). BMC Genom. 16 (1), 1101–1114. 10.1186/s12864-015-2312-8 PMC4690373 26704908

[B49] Zhang J. Yuan H. Li M. Li Y. Wang Y. Ma X. J. (2016). A high-density genetic map of tetraploid *Salix matsudana* using specific length amplified fragment sequencing (SLAF-seq). Plos One 11 (6), e0157777. 10.1371/journal.pone.0157777 27327501 PMC4915623

[B50] Zhao Z. Gu H. Sheng X. Yu H. Wang J. Huang L. (2016). Genome-wide single-sucleotide polymorphisms discovery and high-density genetic map construction in cauliflower using specific-locus amplified fragment sequencing. Front. Plant Sci. 7, 334. 10.3389/fpls.2016.00334 27047515 PMC4800193

[B51] Zhong Y. J. Zhou Y. Y. Li J. X. Yu T. Wu T. Q. Luo J. N. (2017). A high-density linkage map and QTL mapping of fruit-related traits in pumpkin (*Cucurbita moschata* Duch.). Sci. Rep. 7 (1), 12785. 10.1038/s41598-017-13216-3 28986571 PMC5630576

[B52] Zhou H. W. Lu B. Y. Ma H. Y. Gao P. Luan F. S. Gao Q. F. (2016). QTL mapping of watermelon seed traits. Acta Hort. Sinica 43 (4), 715–723. 10.16420/j.issn.0513-353x.2015-0973

[B53] Zhu W. Y. Huang L. Chen L. Yang J. T. Wu J. N. Qu M. L. (2016). A high-density genetic linkage map for cucumber (*Cucumis sativus* L.): based on specific length amplified fragment (SLAF) sequencing and QTL analysis of fruit traits in cucumber. Front. Plant Sci. 7 (569), 437. 10.3389/fpls.2016.00437 27148281 PMC4835494

[B54] Zhu Y. (2015)., in: Construction of genetic linkage maps using specific length amplified fragment markers and identification of a quantitative trait locus for anthracnose resistance in walnut (Juglans regia), Shandong Agricultural University Shandong, China. 10.1186/s12864-015-1822-8 PMC4539690 26283231

[B55] Zraidi A. Pachner M. Shojaeiyan A. Gong L. Lelley T. (2007). A consensus map for *Cucurbita pepo* . Mol. Breed. 20 (4), 375–388. 10.1007/s11032-007-9098-6

