# Supplementary figures and images for: Construction of a High-Density Genetic Map and Analysis of Seed-Related Traits Using Specific Length Amplified Fragment Sequencing for Cucurbita maxima
Source: Front Plant Sci. 2020 Feb 21;10:1782. doi: 10.3389/fpls.2019.01782 (PMC7046561; doi:10.3389/fpls.2019.01782)

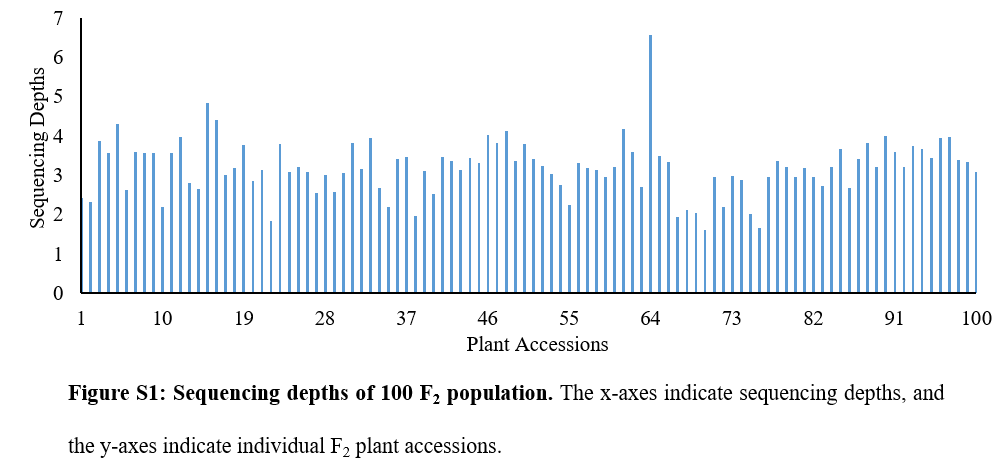

Supplement: Supplementary file 1 [file Image_1.png]

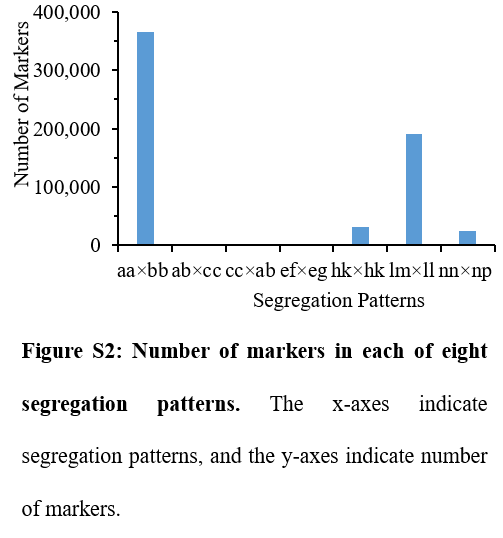

Supplement: Supplementary file 2 [file Image_2.png]

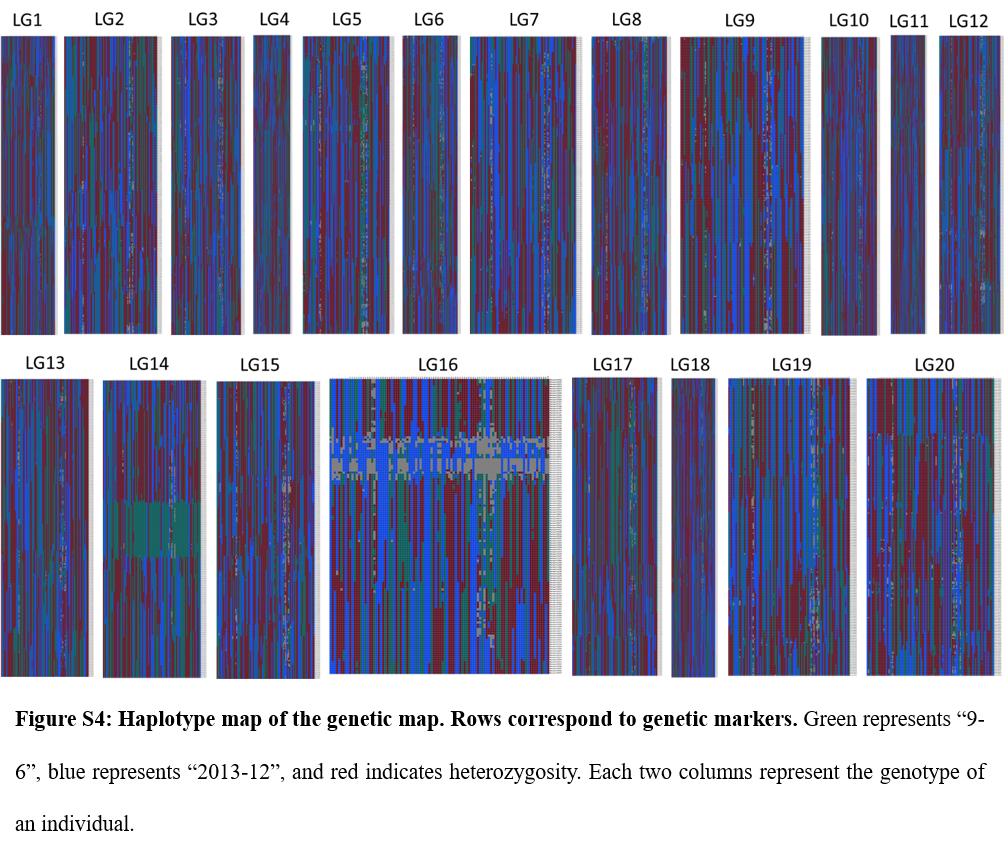

Supplement: Supplementary file 4 [file Image_4.png]

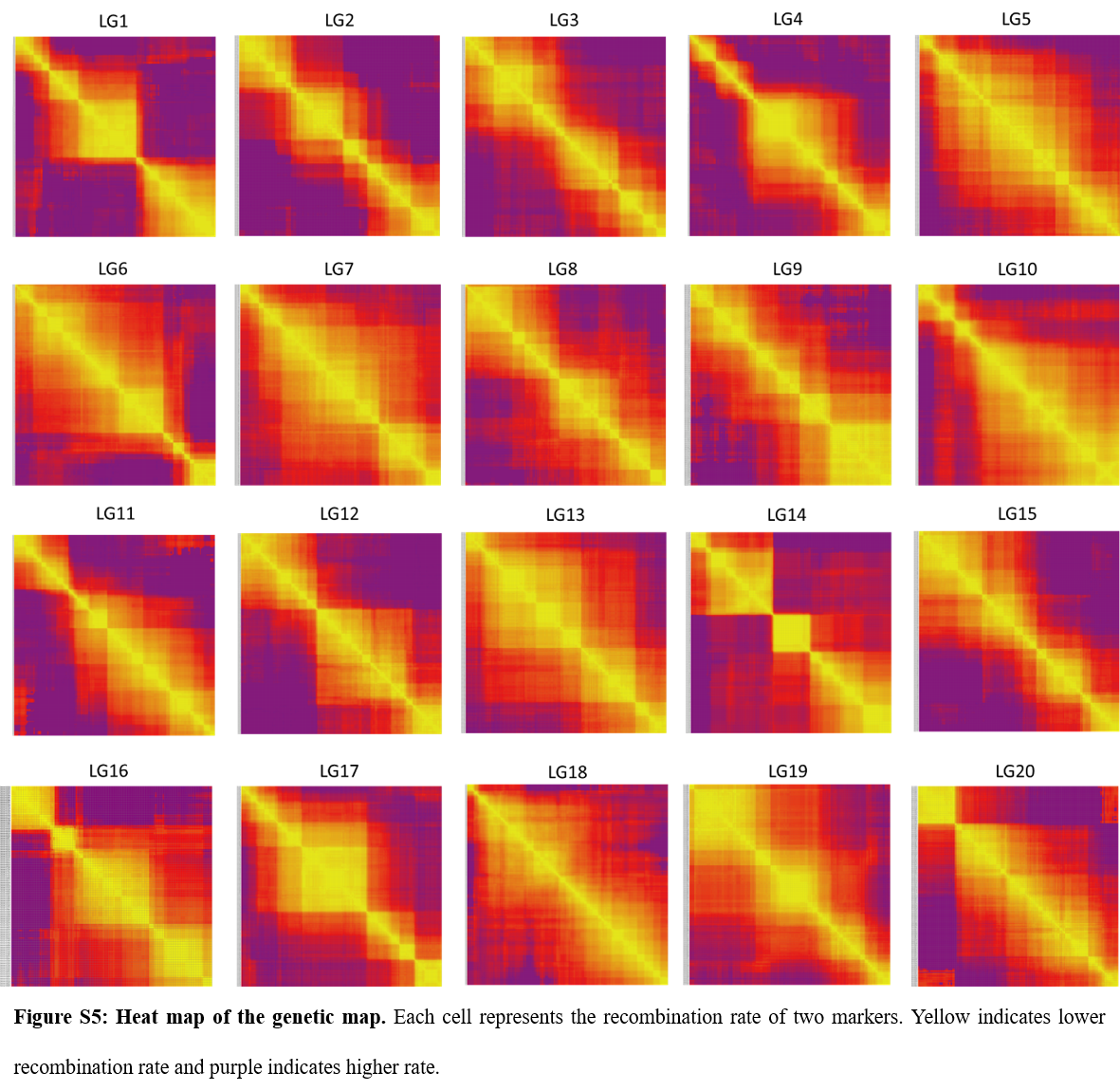

Supplement: Supplementary file 5 [file Image_5.png]

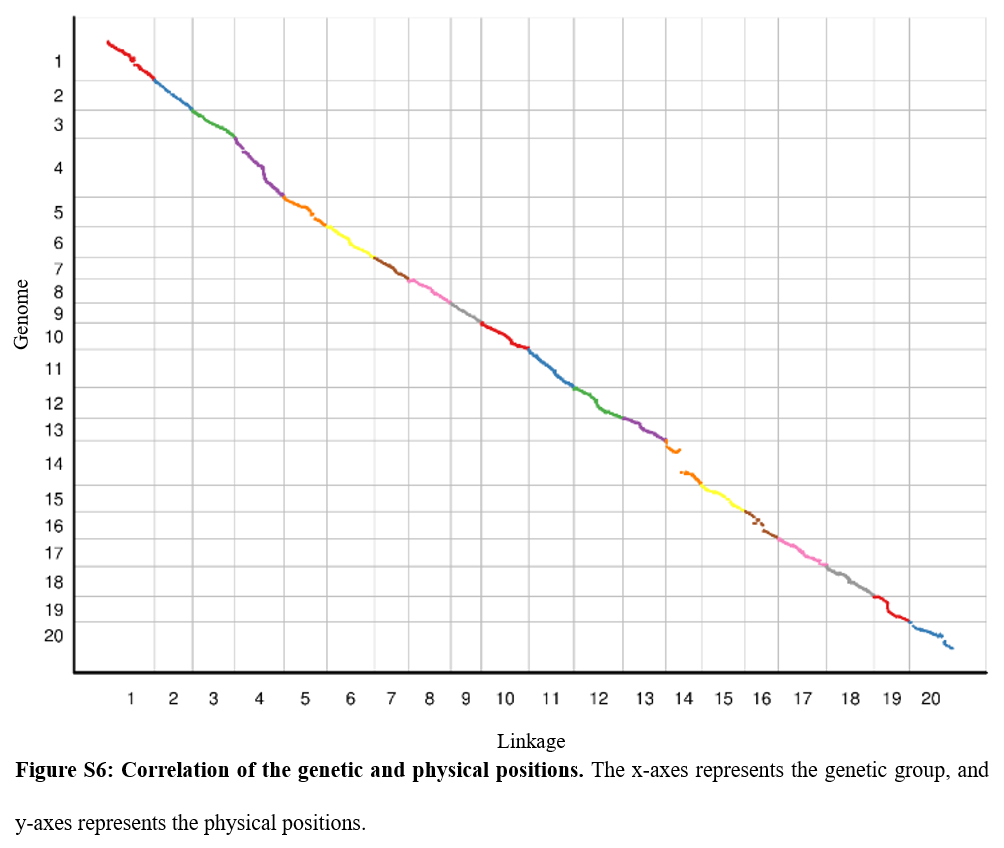

Supplement: Supplementary file 6 [file Image_6.png]

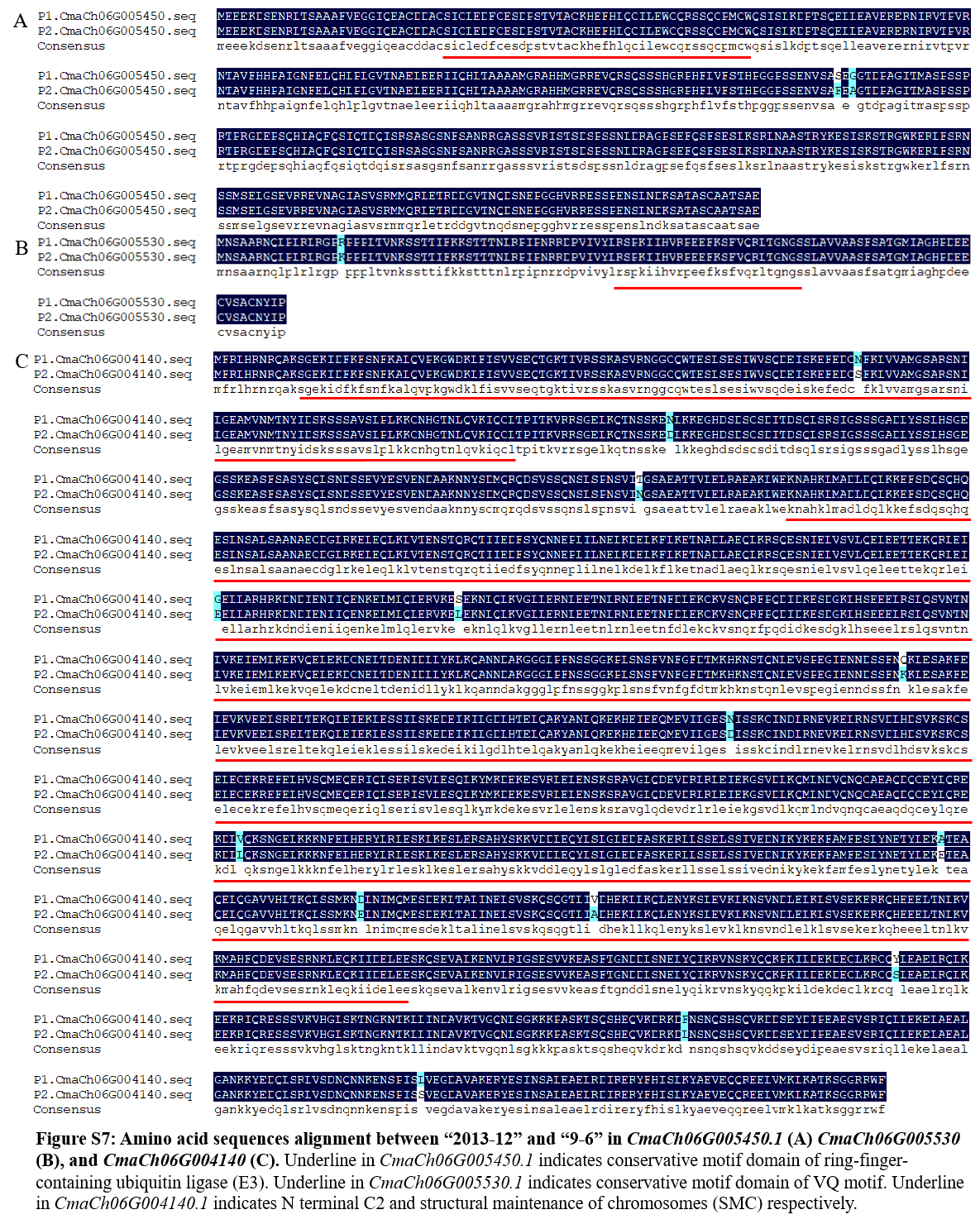

Supplement: Supplementary file 7 [file Image_7.png]
