# Supplementary material for: Construction of a High-Density Genetic Map and Analysis of Seed-Related Traits Using Specific Length Amplified Fragment Sequencing for Cucurbita maxima
Source: Front Plant Sci. 2020 Feb 21;10:1782. doi: 10.3389/fpls.2019.01782 (PMC7046561; doi:10.3389/fpls.2019.01782)
Supplement: Supplementary file 8 [file Table_1.docx]

| Primer name | primer sequence (5′-3′) | product lenth (bp) | restriction site (bp) | Enzyme |
| --- | --- | --- | --- | --- |
| M1047465 | Fwd: GACAGCATCCTGATTCTCGG | 888 | 420 | *Xho* I |
|  | Rev: GATTTCTTGGACGGTGGGTA |  |  |  |
| M1468248 | Fwd: AGAGCCGATGACACGCACA | 620 | 206 | *Eco*R I |
|  | Rev: GTCCACACCGTCCTCAATAATG |  |  |  |
| M1929605 | Fwd: ATGAGGCGTCAATGTGGGA | 585 | 102 | *Sac* I |
|  | Rev: GTTGAATCTATCCCGAACCTCC |  |  |  |
| M2110463 | Fwd: GTTGTCGTGTTTTGACGCCTG | 520 | 150 | *Xho* I |
|  | Rev: ATTCCAATGCTATGTTCCTCCA |  |  |  |
| M2374213 | Fwd: ATGTCGCCAGCCAAGAACC | 546 | 216 | *Sac* I |
|  | Rev: ACGAACCCACCAATGGAGAA |  |  |  |
| M2802290 | Fwd: TATGCTAACAGTGGACTTGGG | 690 | 308 | *Xho* I |
|  | Rev: CTTGTGTCGGGGAATAATAGA |  |  |  |
| M2984727 | Fwd: AGGTTCGGTTATCCGCTGAC | 669 | 426 | *Sal* I |
|  | Rev: TTCTTTGGTGTTCGTATTTTGTTC |  |  |  |
| M3507675 | Fwd: TCTTGAACCTATCCTCTACG | 192 | 32 | *Eco*R I |
|  | Rev: GAGGGTTTTATGCTCAGTTA |  |  |  |
| M3950547 | Fwd: TTTCACAAACTCCAATCAC | 345 | 94 | *Eco*R I |
|  | Rev: TTAGAGAAGAGGACATAAGACT |  |  |  |
| M4491087 | Fwd: TCATTGTTGGCTGCGTCG | 615 | 216 | *Hin*d III |
|  | Rev: AGAGCATTTGGGCGTTCCT |  |  |  |
| M4930644 | Fwd: GGGGACATCAGGAGGA | 449 | 165 | *Eco*R I |
|  | Rev: CAACCATTTGAGTGCCA |  |  |  |

Table S1. Primers used for CAPS markers
