# Supplementary material for: Construction of a High-Density Genetic Map and Analysis of Seed-Related Traits Using Specific Length Amplified Fragment Sequencing for Cucurbita maxima
Source: Front Plant Sci. 2020 Feb 21;10:1782. doi: 10.3389/fpls.2019.01782 (PMC7046561; doi:10.3389/fpls.2019.01782)
Supplement: Supplementary file 11 [file Table_4.docx]

Table S4. SNP and SLAF number in 20 LGs

| LG ID | SNP Number | SLAF Number |
| --- | --- | --- |
| LG 01 | 59,712 | 28,453 |
| LG 02 | 45,839 | 21,769 |
| LG 03 | 39,677 | 20,707 |
| LG 04 | 77,881 | 43,545 |
| LG 05 | 58,552 | 23,302 |
| LG 06 | 46,321 | 21,087 |
| LG 07 | 34,103 | 17,576 |
| LG 08 | 29,881 | 17,334 |
| LG 09 | 43,997 | 19,622 |
| LG 10 | 54,567 | 20,066 |
| LG 11 | 66,712 | 29,144 |
| LG 12 | 55,782 | 22,665 |
| LG 13 | 45,283 | 19,281 |
| LG 14 | 61,246 | 30,667 |
| LG 15 | 37,084 | 19,492 |
| LG 16 | 42,254 | 21,937 |
| LG 17 | 49,404 | 20,592 |
| LG 18 | 63,769 | 22,964 |
| LG 19 | 46,036 | 20,203 |
| LG 20 | 49,172 | 20,145 |
| others | 190,116 | 124,443 |
| Total | 1,197,388 | 584,994 |

SNP and SLAF number in 20 LGs. Number of the *C. maxima* in each LG is also shown.
