# Supplementary material for: Construction of a High-Density Genetic Map and Analysis of Seed-Related Traits Using Specific Length Amplified Fragment Sequencing for Cucurbita maxima
Source: Front Plant Sci. 2020 Feb 21;10:1782. doi: 10.3389/fpls.2019.01782 (PMC7046561; doi:10.3389/fpls.2019.01782)
Supplement: Supplementary file 14 [file Table_7.docx]

Table S7. Summary of marker depths on the genetic linkage map

| Sample | Marker Number | Total Depth | Average Depth |
| --- | --- | --- | --- |
| 2013-12 | 8,406 | 134,754 | 16.03 |
| 9-6 | 8,406 | 148,231 | 17.63 |
| Offsping | 8,247 | 216,408 | 26.24 |

Marker number, total depth and average depth of two parents and offspring on the genetic linkage map are shown.
