# Supplementary material for: Construction of a High-Density Genetic Map and Analysis of Seed-Related Traits Using Specific Length Amplified Fragment Sequencing for Cucurbita maxima
Source: Front Plant Sci. 2020 Feb 21;10:1782. doi: 10.3389/fpls.2019.01782 (PMC7046561; doi:10.3389/fpls.2019.01782)
Supplement: Supplementary file 15 [file Table_8.docx]

Table S8. Marker depths in parents and F_2_ population on the genetic linkage map

| Sample ID | Marker Number | Total  Depth | Average  Depth | Sample ID | Marker Number | Total  Depth | Average  Depth |
| --- | --- | --- | --- | --- | --- | --- | --- |
| 2013-12 | 8406 | 134754 | 16.03 | F_2_-50 | 8389 | 293853 | 35.03 |
| 9-6 | 8406 | 148231 | 17.63 | F_2_-51 | 8365 | 294298 | 35.18 |
| F_2_-1 | 8296 | 129945 | 15.66 | F_2_-52 | 8367 | 252070 | 30.13 |
| F_2_-2 | 8146 | 104976 | 12.89 | F_2_-53 | 8314 | 317412 | 38.18 |
| F_2_-3 | 8350 | 246831 | 29.56 | F_2_-54 | 8349 | 185175 | 22.18 |
| F_2_-4 | 8258 | 139073 | 16.84 | F_2_-55 | 8306 | 221540 | 26.67 |
| F_2_-5 | 8328 | 216521 | 26.00 | F_2_-56 | 8323 | 227997 | 27.39 |
| F_2_-6 | 8350 | 157978 | 18.92 | F_2_-57 | 8374 | 179604 | 21.45 |
| F_2_-7 | 8369 | 223922 | 26.76 | F_2_-58 | 8379 | 163330 | 19.49 |
| F_2_-8 | 8365 | 215584 | 25.77 | F_2_-59 | 8384 | 239714 | 28.59 |
| F_2_-9 | 8391 | 227314 | 27.09 | F_2_-60 | 8404 | 217457 | 25.88 |
| F_2_-10 | 8205 | 126910 | 15.47 | F_2_-61 | 8379 | 229966 | 27.45 |
| F_2_-11 | 8322 | 247222 | 29.71 | F_2_-62 | 8382 | 209305 | 24.97 |
| F_2_-12 | 8303 | 245647 | 29.59 | F_2_-63 | 8370 | 197173 | 23.56 |
| F_2_-13 | 8256 | 141967 | 17.20 | F_2_-64 | 8384 | 265919 | 31.72 |
| F_2_-14 | 8262 | 169118 | 20.47 | F_2_-65 | 8390 | 277764 | 33.11 |
| F_2_-15 | 8350 | 292093 | 34.98 | F_2_-66 | 8344 | 201130 | 24.10 |
| F_2_-16 | 8345 | 253215 | 30.34 | F_2_-67 | 8388 | 428849 | 51.13 |
| F_2_-17 | 8246 | 151748 | 18.40 | F_2_-68 | 8390 | 275335 | 32.82 |
| F_2_-18 | 8216 | 201439 | 24.52 | F_2_-69 | 8392 | 226646 | 27.01 |
| F_2_-19 | 8267 | 196610 | 23.78 | F_2_-70 | 8328 | 113348 | 13.61 |
| F_2_-20 | 7321 | 163362 | 22.31 | F_2_-71 | 7828 | 63780 | 8.15 |
| F_2_-21 | 6006 | 154277 | 25.69 | F_2_-72 | 7242 | 44284 | 6.11 |
| F_2_-22 | 8303 | 136851 | 16.48 | F_2_-73 | 7728 | 56191 | 7.27 |
| F_2_-23 | 8381 | 285651 | 34.08 | F_2_-74 | 6170 | 24382 | 3.95 |
| F_2_-24 | 8370 | 207903 | 24.84 | F_2_-75 | 8246 | 109099 | 13.23 |
| F_2_-25 | 8379 | 256690 | 30.63 | F_2_-76 | 7916 | 88229 | 11.15 |
| F_2_-26 | 8383 | 239898 | 28.62 | F_2_-77 | 6814 | 28790 | 4.23 |
| F_2_-27 | 8356 | 174661 | 20.90 | F_2_-78 | 7834 | 68296 | 8.72 |
| F_2_-28 | 8324 | 239880 | 28.82 | F_2_-79 | 8181 | 92069 | 11.25 |
| F_2_-29 | 8325 | 192466 | 23.12 | F_2_-80 | 8378 | 242364 | 28.93 |
| F_2_-30 | 8345 | 223651 | 26.80 | F_2_-81 | 8376 | 249579 | 29.80 |
| F_2_-31 | 8379 | 301463 | 35.98 | F_2_-82 | 8372 | 242233 | 28.93 |
| F_2_-32 | 8353 | 228026 | 27.30 | F_2_-83 | 8391 | 230896 | 27.52 |
| F_2_-33 | 8387 | 299039 | 35.66 | F_2_-84 | 8362 | 245470 | 29.36 |
| F_2_-34 | 8361 | 202631 | 24.24 | F_2_-85 | 8383 | 225475 | 26.90 |
| F_2_-35 | 8321 | 147263 | 17.70 | F_2_-86 | 8325 | 199677 | 23.99 |
| F_2_-36 | 8378 | 269039 | 32.11 | F_2_-87 | 8360 | 237276 | 28.38 |
| F_2_-37 | 8370 | 247285 | 29.54 | F_2_-88 | 8396 | 292803 | 34.87 |
| F_2_-38 | 8288 | 154239 | 18.61 | F_2_-89 | 8368 | 207795 | 24.83 |
| F_2_-39 | 8365 | 255895 | 30.59 | F_2_-90 | 8386 | 265777 | 31.69 |
| Sample ID | Marker Number | Total  Depth | Average  Depth | Sample ID | Marker Number | Total  Depth | Average  Depth |
| F_2_-40 | 8380 | 187460 | 22.37 | F_2_-91 | 8383 | 249847 | 29.80 |
| F_2_-41 | 8396 | 288674 | 34.38 | F_2_-92 | 8375 | 302163 | 36.08 |
| F_2_-42 | 8374 | 254974 | 30.45 | F_2_-93 | 8389 | 273949 | 32.66 |
| F_2_-43 | 8391 | 244063 | 29.09 | F_2_-94 | 8387 | 224505 | 26.77 |
| F_2_-44 | 8346 | 250998 | 30.07 | F_2_-95 | 8374 | 288349 | 34.43 |
| F_2_-45 | 8377 | 255199 | 30.46 | F_2_-96 | 8387 | 284710 | 33.95 |
| F_2_-46 | 8397 | 316389 | 37.68 | F_2_-97 | 8383 | 251013 | 29.94 |
| F_2_-47 | 8381 | 299924 | 35.79 | F_2_-98 | 8386 | 301837 | 35.99 |
| F_2_-48 | 8383 | 306743 | 36.59 | F_2_-99 | 8392 | 254255 | 30.30 |
| F_2_-49 | 8359 | 255384 | 30.55 | F_2_-100 | 8374 | 249724 | 29.82 |
| Offspring  average | 824691 | 21640793 | 26.24 |  |  |  |  |

Marker number, total depth and average depth of each sequencing sample on the genetic linkage map is shown.
