# Supplementary material for: Construction of a High-Density Genetic Map and Analysis of Seed-Related Traits Using Specific Length Amplified Fragment Sequencing for Cucurbita maxima
Source: Front Plant Sci. 2020 Feb 21;10:1782. doi: 10.3389/fpls.2019.01782 (PMC7046561; doi:10.3389/fpls.2019.01782)
Supplement: Supplementary file 16 [file Table_9.docx]

Supplementary Table S9. The Spearman correlation coefficients of each LGs

| Linkage Group ID | Spearman | Linkage Group ID | Spearman |
| --- | --- | --- | --- |
| LG 01 | 0.99 | LG 11 | 1.00 |
| LG 02 | 1.00 | LG 12 | 0.99 |
| LG 03 | 1.00 | LG 13 | 1.00 |
| LG 04 | 1.00 | LG 14 | 0.98 |
| LG 05 | 1.00 | LG 15 | 1.00 |
| LG 06 | 1.00 | LG 16 | 0.99 |
| LG 07 | 1.00 | LG 17 | 0.99 |
| LG 08 | 0.99 | LG 18 | 1.00 |
| LG 09 | 1.00 | LG 19 | 0.98 |
| LG 10 | 1.00 | LG 20 | 0.99 |

The Spearman correlation coefficients of each linkage group is shown. Number of the *C. maxima* in each LG is also shown.
